# Early-pregnancy transcriptome signatures of preeclampsia: from peripheral blood to placenta

**DOI:** 10.1038/s41598-020-74100-1

**Published:** 2020-10-12

**Authors:** Aishwarya P. Yadama, Enrico Maiorino, Vincent J. Carey, Thomas F. McElrath, Augusto A. Litonjua, Joseph Loscalzo, Scott T. Weiss, Hooman Mirzakhani

**Affiliations:** 1 Channing Division of Network Medicine, Department of Medicine, Brigham and Women’s Hospital, Harvard Medical School, Boston, MA USA; 2 Division of Maternal Fetal-Medicine, Department of Obstetrics and Gynecology, Brigham and Women’s Hospital, Harvard Medical School, Boston, MA USA; 3 Division of Pediatric Pulmonary Medicine, Golisano Children’s Hospital at University of Rochester Medical Center, Rochester, NY USA; 4 Department of Medicine, Brigham and Women’s Hospital, Harvard Medical School, Boston, MA USA

**Keywords:** Computational biology and bioinformatics, Systems biology

## Abstract

Several studies have linked maternal asthma, excess BMI, and low vitamin D status with increased risk of Preeclampsia (PE) development. Given prior evidence in the literature and our observations from the subjects in the Vitamin D Antenatal Asthma Reduction Trial (VDAART), we hypothesized that PE, maternal asthma, vitamin D insufficiency, and excess body mass index (BMI) might share both peripheral blood and placental gene signatures that link these conditions together. We used samples collected in the VDAART to investigate relationships between these four conditions and gene expression patterns in peripheral blood obtained at early pregnancy. We identified a core set of differentially expressed genes in all comparisons between women with and without these four conditions and confirmed them in two separate sets of samples. We confirmed the differential expression of the shared gene signatures in the placenta from an independent study of preeclampsia cases and controls and constructed the preeclampsia module using protein–protein interaction networks. CXC chemokine genes showed the highest degrees of connectivity and betweenness centrality in the peripheral blood and placental modules. The shared gene signatures demonstrate the biological pathways involved in preeclampsia at the pre-clinical stage and may be used for the prediction of preeclampsia.

## Introduction

Preeclampsia (PE) is a complex pregnancy disorder that can affect up to 8% of pregnant women^1–3^. This pregnancy-specific complication accounts for a quarter of all maternal mortality^1^ and causes substantial maternal and prenatal morbidity^4^. Because the definitive etiopathogenesis of PE is not yet understood, preventive measures are scarce (i.e., Aspirin treatment)^5^, and the only definitive treatment is delivery of the fetus. Previous investigations have identified multiple risk factors for PE, a closer exploration of which might aid in identifying the responsible biological pathways and help us identify novel strategies for its primary and secondary prevention. Several studies and systematic reviews of these studies demonstrated that maternal asthma^6^, vitamin D insufficiency^7^, and excess body mass index (BMI)^8,9^ during pregnancy are risk factors for PE. Results from other investigations suggest these risk factors might interact and share pathobiological pathways. Excess BMI has been linked to asthma^10–12^, a pro-inflammatory environment during pregnancy, both systemically and in the placenta^13^, and modulation of bioavailability of vitamin D^14^. Finally, vitamin D status also affects maternal asthma activity during pregnancy^15^ and contributes directly to PE risk^16,17^.

The Vitamin D Antenatal Asthma Reduction Trial (VDAART) was a randomized, double-blind, placebo-controlled clinical trial of vitamin D supplementation (4400 versus 400 International Units Vitamin D daily) in pregnant women to prevent the development of pregnancy complications such as PE and asthma or atopy in their children by the age of 3 years old. Ancillary studies conducted using data from this trial corroborate the evidence that maternal asthma, particularly lack of asthma control, vitamin D (25-hydroxyvitamin D [25OHD]) deficiency, and excess BMI are risk factors for PE^2,3,18–20^. Findings from these studies also demonstrated the interplay between maternal asthma, BMI, and vitamin D levels during pregnancy, particularly in early pregnancy ^21^. Abnormal placentation and immune interactions at the maternal–fetal interface have been recognized as the root cause of PE^22^; however, the onset, severity, and progression of the disease are significantly affected by maternal risk factors and response to molecules released from the placenta. Direct exploration of dysregulation in the biological processes related to PE is not easily studied at early gestational time points owing to limited access to placental tissue in development. However, maternal peripheral blood is accessible at early pregnancy, and blood transcriptional changes could mirror placental processes, providing additional insight into molecular mechanisms occurring in women who develop PE and resulting in an improved predictive capability.

**Figure 1:**
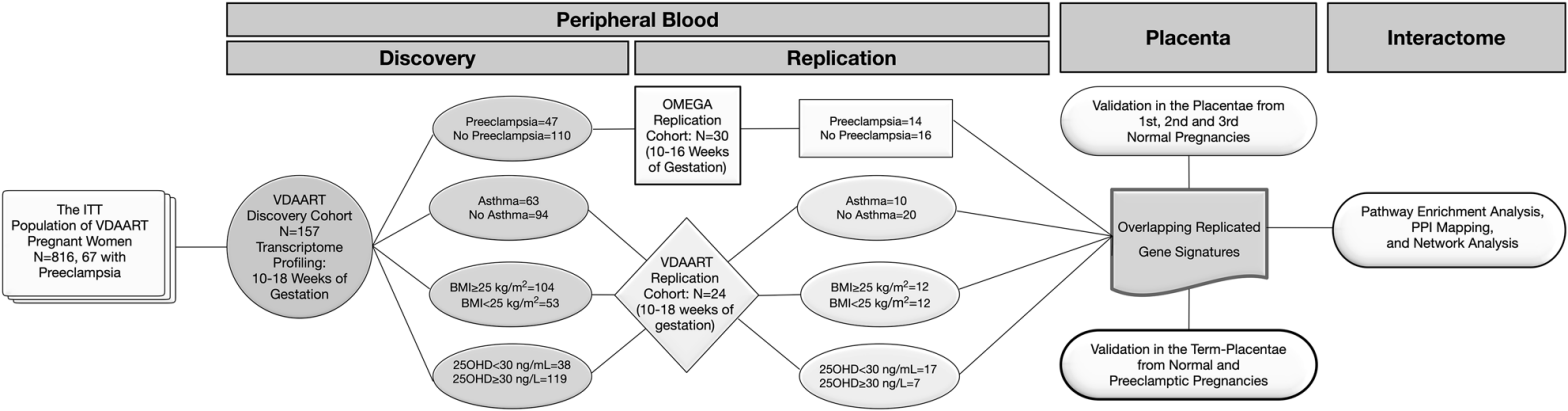
Study flow chart for differential expression in peripheral blood and replication of peripheral blood gene signatures in the placenta. Figure generated using OmniGraffle (https://www.omnigroup.com/omnigraffle).

Given prior evidence in the literature and from the VDAART cohort, we hypothesized that PE, maternal asthma, vitamin D insufficiency, and excess BMI in early pregnancy might share dysregulated biological pathways in peripheral blood and placenta. To investigate this hypothesis, we conducted a microarray differential gene expression study in a nested case–control subset of pregnant women participating in the VDAART trial. We identified a core set of differentially expressed gene signatures associated with each study condition that was confirmed in the early pregnancy of separate sets of pregnant women (Fig. 1). To demonstrate the tissue-specificity of the overlapping gene signatures in the placenta, we examined their differential expression in healthy early-pregnancy placenta and term-placenta of pregnancies with and without PE. Furthermore, we performed a network-based analysis of these overlapping gene signatures to visualize the commonality between these conditions at the molecular level.

## Results

### Study cohort characteristics

The pregnant women in the VDAART discovery cohort (N = 157, 47 with PE) had a mean gestational age of 14 ± 2.66 weeks (10–18 weeks of gestational age [wga]). Among other baseline characteristics of this cohort (Table 1), pregnant women with PE had lower gestational age at delivery than those with uncomplicated pregnancies (37.7 ± 3.5 and 39 ± 1.0; respectively; *P* < 0.001). Vitamin D insufficiency (25OHD < 30 ng/ml) at 10–18 wga was also more frequent among pregnant women with a PE diagnosis compared with controls with no complication during their pregnancies (41/47 vs 78/110; *P* = 0.047). Among 104 pregnant women who had a BMI ≥ 25 kg/m^2^ at early pregnancy, 34 women (33%) had PE during their pregnancies. Among 63 pregnant women with asthma, 20 had PE (32%). However, the distribution of pregnant women with a BMI ≥ 25 kg/m^2^ at early pregnancy and those with asthma were not different between pregnancies with and without PE (Table 1).

**Table 1:** Clinical characteristics of the discovery cohort in VDAART.

	Preeclampsia	No Preeclampsia	P-value
N	47	110	
Maternal Age (mean (SD))	25.78 (4.95)	26.63 (5.08)	0.332
Maternal Age: ≥ 30 Years Old (%)	10 (21.3)	30 (27.3)	0.555
Gestational Age (mean (SD))	13.85 (2.66)	14.25 (2.69)	0.386
Previous Pregnancies vs. First Pregnancy: Previous Pregnancy (%)	26 (55.3)	72 (65.5)	0.307
Gestational Age at Delivery (mean (SD))	37.70 (3.50)	39.15 (1.02)	< 0.001
**Site Name (%)**			0.711
Boston	10 (21.3)	18 (16.4)	
San Diego	15 (31.9)	34 (30.9)	
St. Louis	22 (46.8)	58 (52.7)	
**Maternal Race (%)**			0.11
Black or African American	22 (46.8)	61 (55.5)	
Other	8 (17.0)	7 (6.4)	
White	17 (36.2)	42 (38.2)	
**Maternal Education (%)**			0.213
College graduate	8 (17.0)	36 (32.7)	
Did not graduate from high school	10 (21.3)	15 (13.6)	
High school, technical school	18 (38.3)	37 (33.6)	
Junior college/some college	11 (23.4)	22 (20.0)	
Married/Not Married (Separated or Divorced) (%)	17/30 (36.2/63.8)	38/72 (34.5/65.5)	0.99
**Maternal Income (%)**			0.252
Do not know/prefer not to answer	14 (29.8)	20 (18.2)	
Less than $50,000	20 (42.6)	58 (52.7)	
Over $50,000	13 (27.7)	32 (29.1)	
BMI at Enrollment: ≥ 25 kg/m^2^ (%)	34 (72.3)	70 (63.6)	0.383
Maternal Asthma: Yes (%)	20 (42.6)	43 (39.1)	0.820
Maternal Atopy: Yes (%)	36 (76.6)	87 (79.1)	0.892
**Vitamin D Status at 10–18 Weeks of Gestation**			
Vitamin D level (mean (SD))	19.72 (8.33)	24.37 (14.55)	0.042
Vitamin D Level: Insufficient (%)	41 (87.2)	78 (70.9)	0.047
**Vitamin D Status at 32–38 Weeks of Gestation**			
Vitamin D Level (mean (SD))	30.49 (16.02)	33.64 (17.67)	0.316
Third Trimester Vitamin D Level: Insufficient (%)	18 (42.9)	57 (52.3)	0.391
Intervention Arm: Supplement/Placebo (%)	23/24 (48.9/51.1)	59/51 (53.6/46.4)	0.715

1. BMI: Body Mass Index; 2. < 30 ng/mL.

The participants in the OMEGA cohort (N = 30, 14 with PE), who were used for the replication of differentially expressed (DE) PE gene signatures identified in the discovery VDAART cohort, were mainly white pregnant women (25/30, 12 with PE) with the mean of gestational ages 16 ± 2.0 weeks (11–18 wga). Further details of the OMEGA study population’s characteristics by PE status are published^23^.

The VDAART replication cohort (N = 24) was selected among asthmatic pregnant women who did not have PE or gestational hypertension during pregnancy. The baseline characteristics by asthma status are provided in Table 2. Among those, having a first pregnancy was less common among asthmatic women than non-asthmatic women (1/10 vs 9/14, respectively; *P* = 0.025).

**Table 2:** Clinical characteristics of the VDAART replication cohort.

	Asthma	No Asthma	P value
N	10	14	
Maternal Age: > 30 Years Old (%)	6 (60.0)	5 (35.7)	0.45
Gestational Age at Enrollment (mean (SD))	13.97 (2.77)	15.20 (2.81)	0.30
Previous Pregnancies vs. First Pregnancy: Previous Pregnancy (%)	9 (90.0)	5 (35.7)	0.03
Gestational Age at Delivery (mean (SD))	35.37 (5.97)	34.66 (7.07)	0.80
Site Name (%)			0.71
Boston	6 (60.0)	6 (42.9)	
San Diego	1 (10.0)	2 (14.3)	
St. Louis	3 (30.0)	6 (42.9)	
Maternal Race (%)			0.25
Black or African American	6 (60.0)	4 (28.6)	
White	2 (20.0)	7 (50.0)	
Other	2 (20.0)	3 (21.4)	
College or higher/Not a college graduate (%)	3/7 (30.0/70.0)	7/7 (50.0/50.0)	0.58
Married/Not Married (Separated or Divorced) (%)	4/6 (40.0/60.0)	7/7 (50.0/50.0)	0.95
Maternal Income (%)			0.65
Do not know/prefer not to answer	1 (10.0)	3 (21.4)	
Less than $50,000	6 (60.0)	6 (42.9)	
Over $50,000	3 (30.0)	5 (35.7)	
BMI^1^ at Enrollment ≥ 25 kg/m^2^ (%)	7 (70.0)	7 (50.0)	0.58
Maternal Atopy: Yes (%)	8 (80.0)	9 (64.3)	0.70
Vitamin D Status at 10–18 Weeks of Gestation			
Vitamin D Level (mean (SD))	20.70 (10.89)	26.14 (11.03)	0.24
Vitamin D Level at Enrollment: Insufficient (%)	7 (70.0)	10 (71.4)	1
Vitamin D Status at 32–38 Weeks of Gestation			
Vitamin D Level (mean (SD))	30.68 (10.83)	40.53 (19.69)	0.29
Third Trimester Vitamin D: Insufficient (%)	2 (33.3)	2 (22.2)	1
Intervention Arm: Supplement/Placebo (%)	5/5 (50.0/50.0)	8/6 (57.1/42.9)	1

1. BMI: Body Mass Index.

2. < 30 ng/mL.

### Differential gene expression and overlapping gene signatures

**Figure 2:**
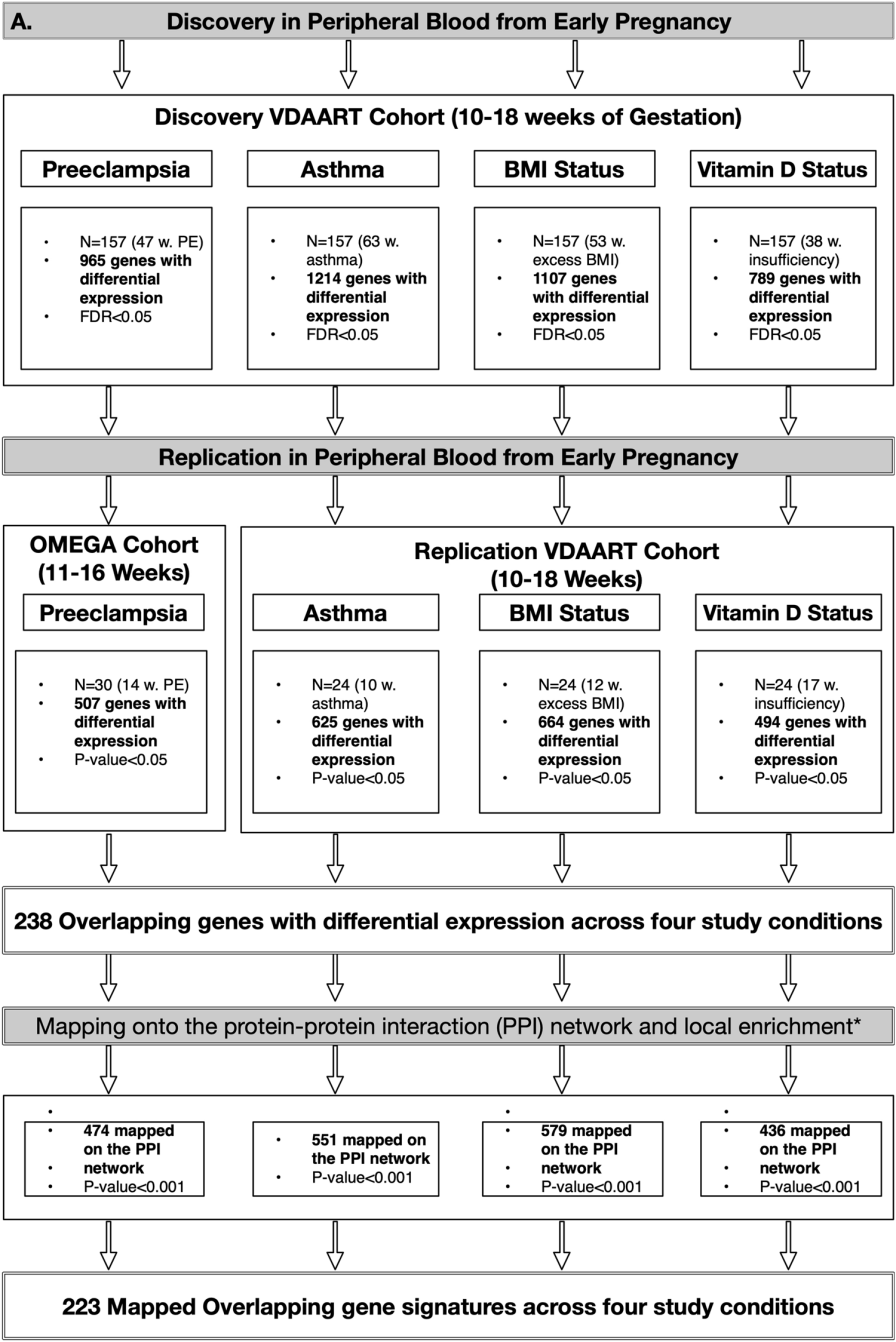
Study workflow and summary of transcriptome profiling’s results. Figure generated using OmniGraffle (https://www.omnigroup.com/omnigraffle).

Figures 1 and 2 summarize the discovery and replication sets as well as the results for both the discovery and replication stages in the study cohorts.

### Discovery cohort

Differential expression analysis of peripheral blood transcriptomes at early pregnancy (10–18 wga) identified 965 genes with differential expressions (463 upregulated) in pregnant women who later developed PE relative to their controls with uncomplicated pregnancies in the discovery cohort (FDR < 0.05). In the same cohort and time point, there were 1,214 (603 upregulated) genes with differential expression between pregnant women with and without asthma (FDR < 0.05). An early-pregnancy BMI ≥ 25 kg/m^2^ was also associated with differential expression of 1,107 genes (608 upregulated) relative to those with a BMI < 25 kg/m^2^ (FDR < 0.05). Vitamin D status of these subjects dichotomized at 30 ng/mL resulted in the differential expression of 789 genes (369 upregulated) associated with early-pregnancy vitamin D insufficiency (25OHD < 30 ng/mL, FDR < 0.05).

### Replication sets

Of 965 genes with differential expression in association with PE status in the discovery cohort, 507 genes (53%) (244 upregulated) were confirmed in the peripheral blood of pregnant women at 11–18 wga who later developed PE relative to their controls (*P* < 0.05) in an extra set of pregnant women from a second cohort. Genes with differential expressions identified in the discovery stage in association with maternal asthma, excess BMI, and vitamin D insufficiency at early pregnancy were confirmed in the peripheral blood of pregnant women at 10–18 wga with no PE complication during their pregnancies. Accordingly, 625 (323 upregulated) out of 1214 DE genes (51%) in association with maternal asthma status were confirmed in the VDAART replication cohort (*P* < 0.05); 664 (363 upregulated) out of 1107 genes (60%) with differential expression related to BMI status were also confirmed in the VDAART replication cohort (*P* < 0.05). Similarly, 494 (215 upregulated) out of 789 of the DE genes (62%) related to vitamin D status were confirmed in the VDAART replication cohort (*P* < 0.05).

**Figure 3:**
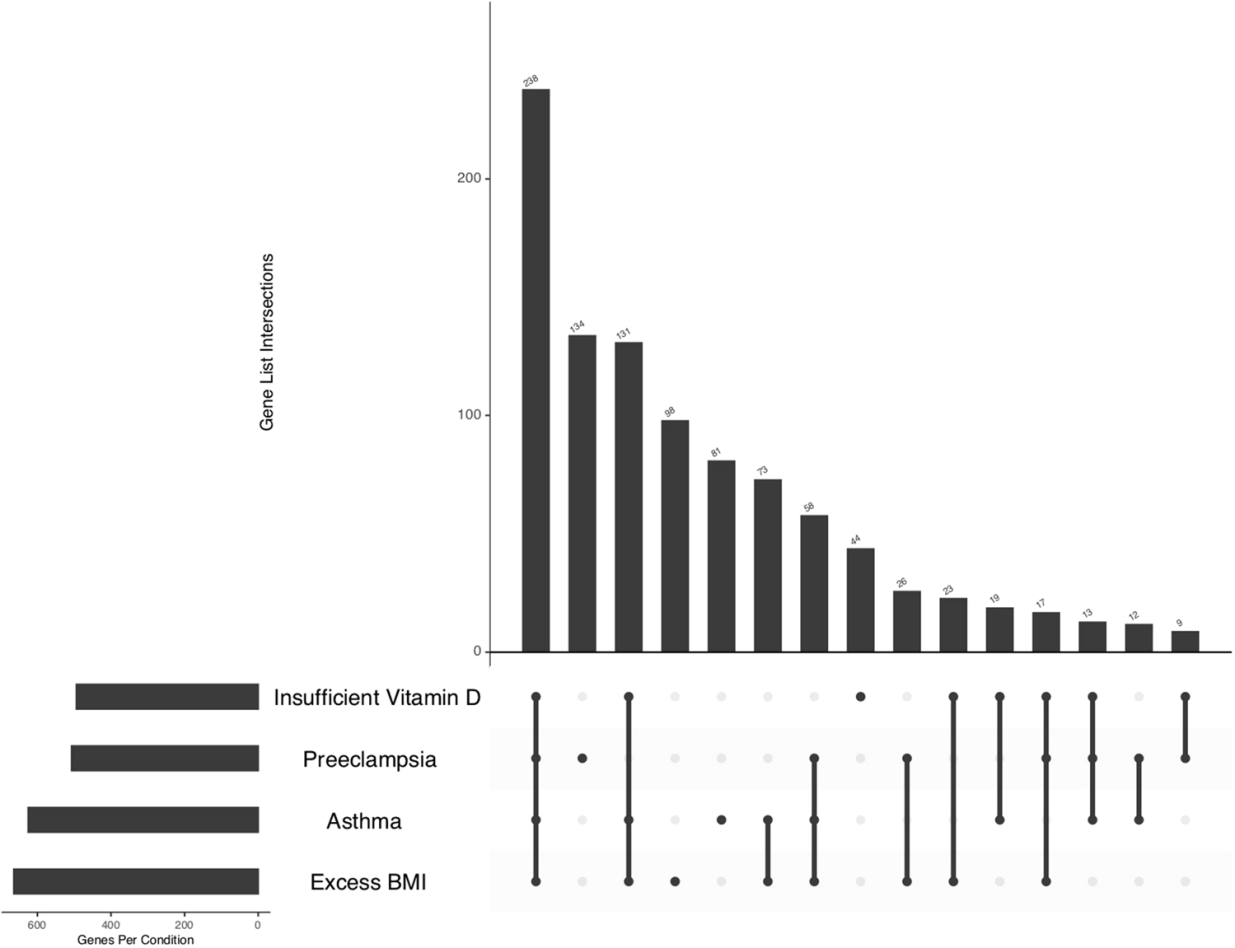
Intersections of Differentially Expressed Genes Across Study Conditions. This bar plot depicts the number of genes at each possible intersection of our study conditions. The first and largest bar represents the intersection of all four study conditions. The second largest bar represents those genes only representing PE. The third bar represents DE genes at the intersection of insufficient vitamin, Asthma, and excess BMI and so forth. The sets are ordered by the size and the combination sets are ordered by the degree. Figure generated using R package “UpSetR” (https://cran.r-project.org/web/packages/UpSetR/vignettes/basic.usage.html).

After replicating the DE genes in association with the four study conditions, we determined the overlapping gene signature across all groups. Accordingly, 238 gene signatures (117 upregulated) comprised the overlapping gene signatures (Supplemental File S2, Table S2A). Figure 3 demonstrates the number of shared and distinct genes across four study conditions. We ascertained that 133 out of 238 genes (55.9% [133/238]) had previously been reported in association with PE in at least one of the 3 resources searched (Supplemental File S2, Table S2A).

### Functional enrichment and similarity of gene set signatures

**Figure 4:**
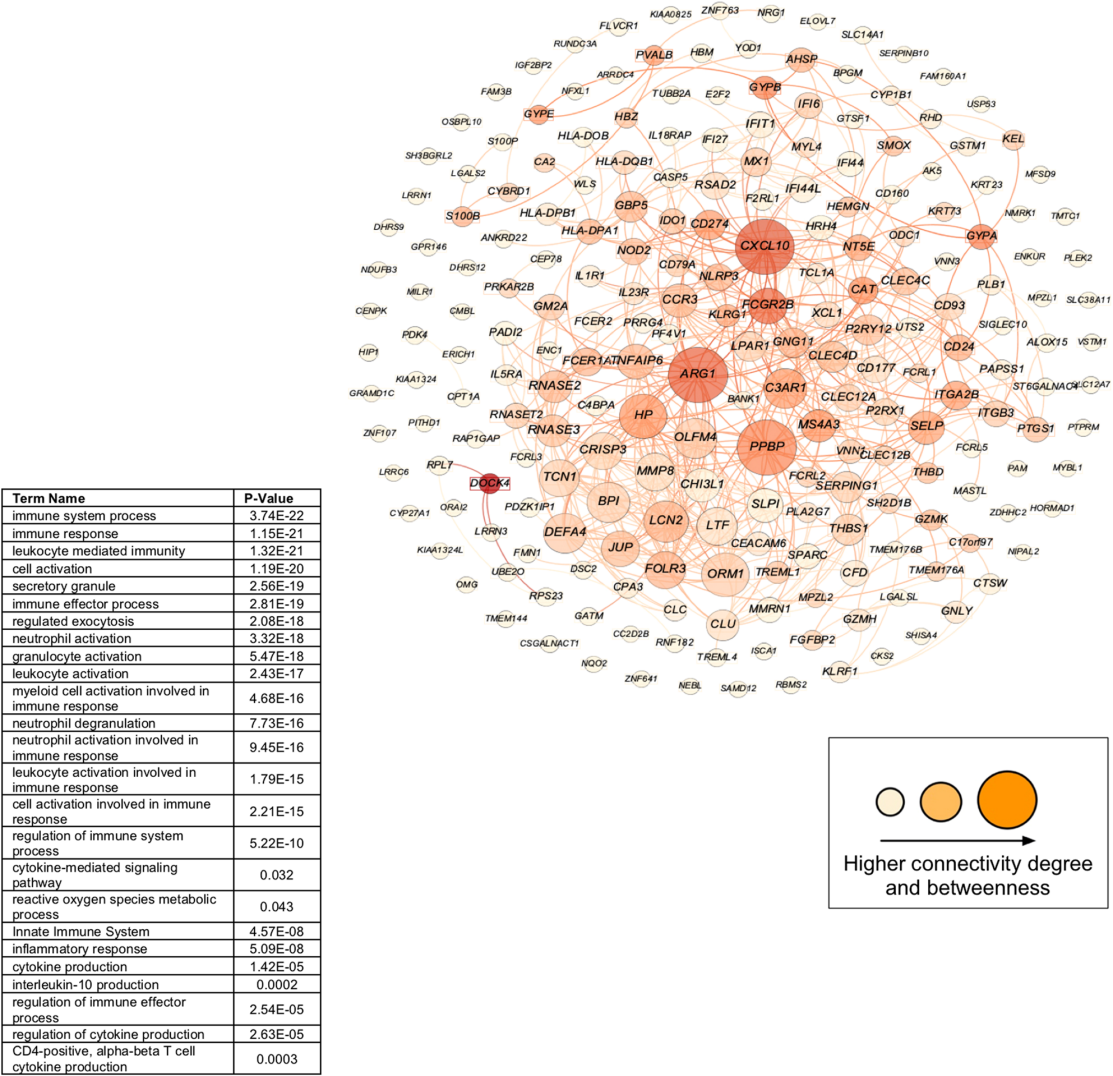
The module (interacting genes in the interactome) of overlapping gene signatures after replication (N = 238) across the study conditions in early-pregnancy peripheral blood and the enriched biological processes. Figure generated using Cytoscape version 3.7.2 (https://cytoscape.org/index.html).

Functional terms that were enriched for asthma, vitamin D and BMI gene signature sets demonstrated high GO semantic similarity measures with PE gene signature sets (> 0.90 for all comparisons between the sets), suggesting the core similarity function of enriched biological and molecular pathways in the four sets of gene signatures. Two hundred and seven of 238 overlapping gene signatures (87%) of the four sets of replicated DE genes (PE, asthma, BMI and vitamin D gene signatures) were mapped to their known GO functional terms. GO enrichment analysis linked the 238 overlapping gene signatures to several immunologic functions including immune system process, response, and regulation (*N* = 103, 85, and 55, respectively); innate and adaptive immune response (*N* = 39 and 13, respectively); inflammatory response (*N* = 33); neutrophil-mediated immunity (*N* = 37); regulation of cytokine and T-cell activation (*N* = 28 and 14 respectively); cytokine-mediated signaling pathway (*N* = 23); interleukin-10 (IL-10) production; and reactive oxygen species metabolic process (*N* = 13), all with a corrected *P-*value < 0.05; Fig. 4, Supplemental File S2, Table S2B).

### Direct interaction of transcriptome signatures in the interactome and their overlap

After replication, each of the four sets of gene signatures was individually mapped onto the interactome and demonstrated enrichment and local clustering in the PPI network (i.e., number of interactions/edges in each set of mapped gene signatures vs. expected number of interactions/edges if the same number of genes/nodes were selected at random from the interactome, individual *P-*value for PPI enrichment < 0.0001 for each study conditions). Accordingly, among the PE gene signatures, 474 (93.49% [474/507]) were mapped onto the PPI network. The number of mapped genes for the gene signatures associated with maternal asthma, BMI and vitamin D status were 551 (88.16% [551/625]), 579 (87.20% [579/664]), and 436 (88.26% [436/494]), respectively.

Of the 238 overlapping gene signatures, 223 (93.70%) genes were mapped onto the interactome and comprised the overlapping gene module (*P* for PPI enrichment < 0.0001). The members (nodes) of the module demonstrated an internal average degree of 5 (529 direct interactions) with an average local clustering coefficient of 0.43 in the PPI network, as a measure of how complete the neighborhood of a module node was.

Of the 223 genes in the overlapping module, 155 nodes had direct interactions comprising the largest connected component (LCC), namely, the “observable overlapping module” (Fig. 4, Supplemental File S2, Table S2A). The LCC nodes had an average degree of 7 (516 direct interactions) with an average local clustering coefficient of 0.60. Among the LCC genes, *CXCL10*, *ARG1*, *PPBP (CXCL7)*, *HP*, *CD274, ITGA2B, ORM1*, *CD24, BPI, NLRP3, FCGR2B,* and *OLFM4* showed high degrees of connectivity and betweenness centrality (Fig. 4), and all of these genes have been previously reported in association with PE (Supplemental File S2, Table S2A).

### Sensitivity analysis

We ran a sensitivity analysis to further validate our results by running differential expression analysis on subjects with all four study conditions (N = 15) versus subjects with none of the study conditions (N = 13). 202 of the genes revealed in this analysis appeared in the list of 238 replicate overlapping gene signatures (85% [202/238]) (Supplemental File S2, Table S2C).

### Closeness of PE module to maternal asthma, BMI, and Vitamin D modules

On further examination of the mapped gene signatures (modules), we also evaluated their closeness to each other in the PPI network. All modules were significantly close to one another, with all Z-scores < − 1.65, corresponding to a *P*-value < 0.05. The same was true for the LCC of genes in each module. The distances between the modules are presented in Table 3.

**Table 3:** Average distances between the preeclampsia module, maternal asthma, excess BMI and vitamin D insufficiency modules.

Closeness of modules in the interactome	Preeclampsia module	Maternal asthma module	BMI module	Vitamin D module
**Preeclampsia module**				
Average distance		2.68	2.70	2.68
Random distance ± SD^		2.73 ± 0.005	2.75 ± 0.006	2.74 ± 0.006
Z-score		− 10.62	− 9.91	− 10.43
**Maternal asthma module**				
Average distance	2.68		2.69	2.67
Random distance ± SD	2.73 ± 0.005		2.75 ± 0.005	2.74 ± 0.006
Z-score	− 10.62		− 11.52	− 11.27
**BMI module**				
Average distance	2.70	2.69		2.70
Random distance ± SD	2.75 ± 0.006	2.75 ± 0.005		2.75 ± 0.006
Z-score	− 9.91	− 11.52		− 10.38
**Vitamin D module**				
Average distance	2.68	2.67	2.70	
Random distance ± SD	2.74 ± 0.006	2.74 ± 0.006	2.75 ± 0.006	
Z-score	− 10.43	− 11.27	− 10.38	

^SD: Standard Deviation.

### Uncomplicated pregnancy

**Figure 5:**
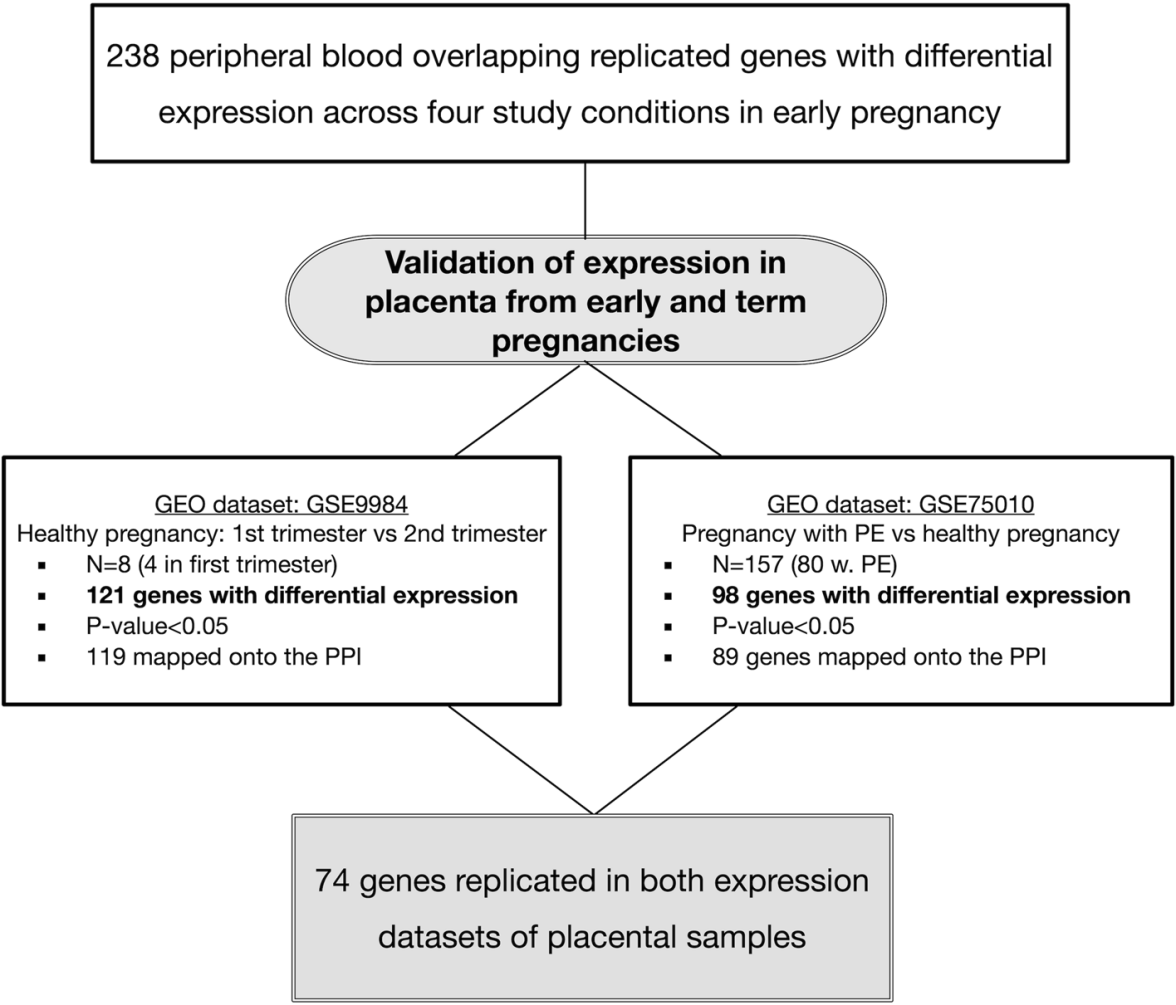
Flow chart of differential expression analysis of the peripheral blood overlapping gene signature in the placenta.

**Figure 6:**
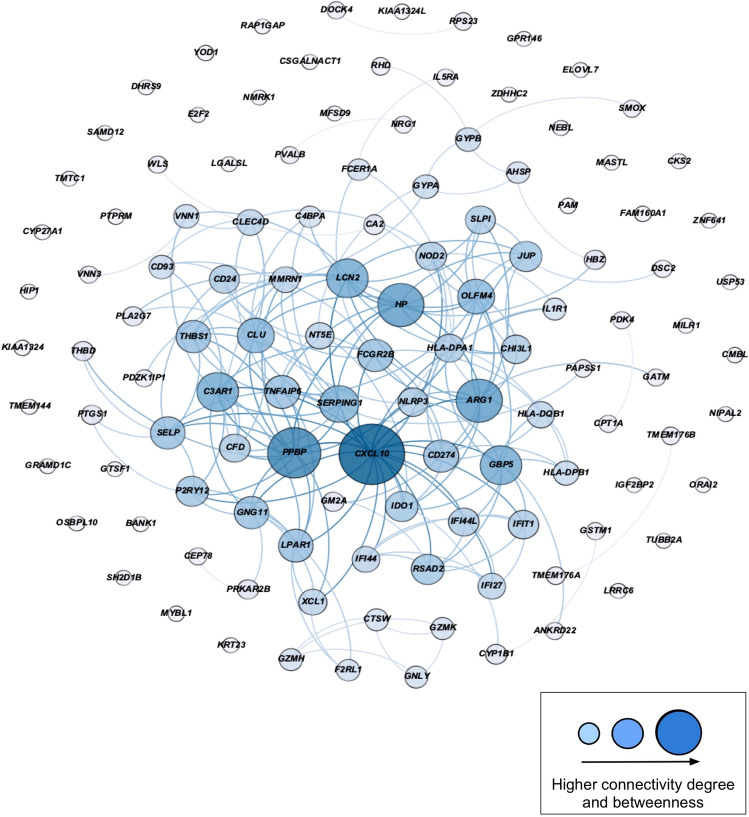
Network depicting a subset of peripheral blood overlapping gene signatures with differential expression interacting in the normal placentae from early pregnancies (1^st^ trimester vs 2^nd^ trimester). Figure generated using Cytoscape version 3.7.2 (https://cytoscape.org/index.html).

In the GEO dataset (GSE9984) from healthy pregnancies, all of the genes in the overlapping signature module were expressed in the placenta samples from the first, second, and third trimesters (*N* = 238 genes with an expression value in all samples at each trimester). One hundred twenty-one genes out of 238 overlapping signatures in the peripheral blood (50.84%) were differentially expressed between the first and second trimesters; of which 119 were mapped onto the interactome with 67 genes having direct interactions comprising the LCC (Fig. 5, Fig. 6, Table S2D). One hundred and eight genes out of 238 overlapping gene signatures (45.4%) were differentially expressed between the second and third trimesters (*P* < 0.05), of which, 105 were mapped onto the interactome with an LCC of 50 nodes. Of note, 74 genes that were differentially expressed in the first trimester relative to the second trimester were also differentially expressed in the third trimester relative to the second trimester (Fig. 5).

*CPY27A1*, one of the non-LCC nodes in the placenta module with differential expression comparing the 1^st^ and 2^nd^-trimester of uncomplicated pregnancies, was connected to the LCC genes of *CXCL10* and *PPBD* via *NR1H4* (*FXR*), *IL10*, *IL4*, and *IL6*, genes in the neighborhood of the LCC genes. Of note, *CYP27A1* was a member of the replicated DE overlapping gene signatures and was also downregulated in peripheral blood of those pregnant women who later developed PE during their pregnancies.

### Uncomplicated pregnancies and pregnancies with preeclampsia

**Figure 7:**
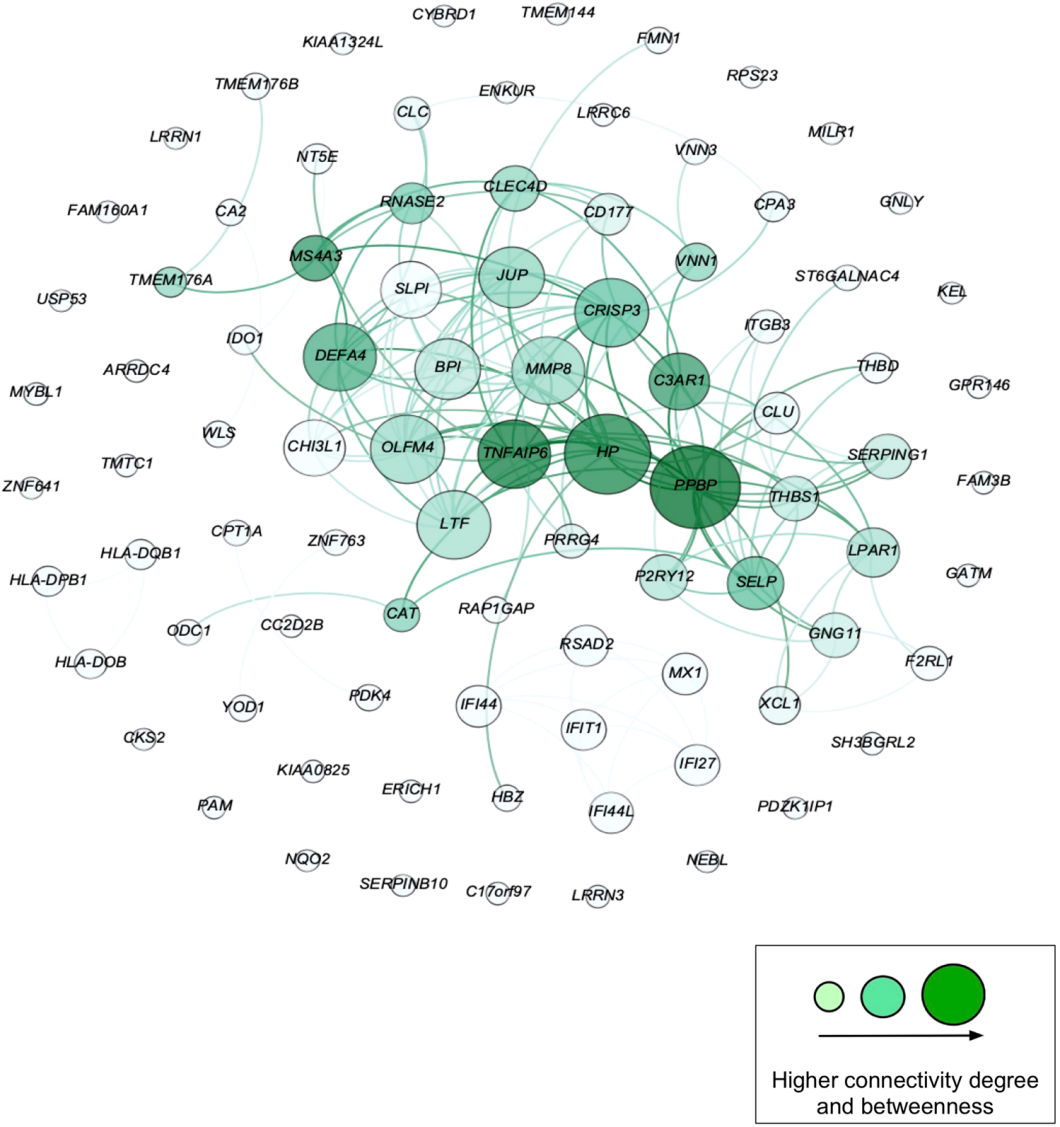
Network depicting a subset of peripheral blood overlapping gene signatures with differential expression interacting in the term placentae from pregnancies with and without preeclampsia. Figure generated using Cytoscape version 3.7.2 (https://cytoscape.org/index.html).

In the second GEO dataset (GSE75010), the genes in the overlapping signature module were expressed in term-placenta samples of healthy pregnancies and those from pregnancies with PE (*N* = 238 genes with an expression value in term-placenta of healthy and preeclamptic pregnancies). Ninety-eight genes out of 238 genes (41%) in the overlapping signatures in peripheral blood were differentially expressed in the term-placenta of pregnancies with PE relative to those from healthy pregnancies (*P* < 0.05, Fig. 5; Supplemental File S2, Table S2E), of which, 89 (91%) were mapped onto the interactome. In this term-placental mapped module, 43 genes comprised the LCC (Fig. 7).

## Discussion

In this study, we carried out transcriptome analysis in whole blood to identify DE gene signatures related to PE in early pregnancy. The PE signatures were confirmed in early pregnancy samples from an independent set of pregnant women and refined according to maternal risk factors, i.e., asthma, excess BMI, and insufficient vitamin D status at early pregnancy. We applied a network approach to construct the interacting gene network based on the PPI network (module) and visualize their connections. We further explored the tissue specificity of these gene signatures in the placenta from preeclamptic and normal pregnancies and ultimately identified the shared signatures (submodule) in peripheral blood and placenta.

### Blood transcriptome provides information on the pre-clinical stage of preeclampsia

Previous investigations conducted in the VDAART cohort (N = 816), as well as external cohorts, revealed that maternal asthma, early-pregnancy excess BMI and vitamin D insufficiency are risk factors for PE development^2,3,6–8,17,18^. Given these results, we felt that the transcriptomic-level analyses on these four study conditions was warranted to investigate biological pathways related to PE at the pre-clinical stage. Blood may be a medium for surrogate information on the proteins expressed in the placenta. Previous investigations conducted gene expression analyses on pregnancies with PE versus those with uncomplicated pregnancies in either peripheral blood or placental tissue^24,25^ mostly at term and delivery; however, few studies have sought to identify gene networks related to PE and its maternal risk factors, i.e., asthma, BMI, and vitamin D status in peripheral blood and placental tissue at early pregnancy^26^.

Our analysis revealed a common whole-blood gene signature associated with PE and the risk of factors of maternal asthma, excess BMI, and vitamin D insufficiency. Sensitivity analysis proved the robustness of this gene signature when compared to genes differentially expressed between subjects with all conditions versus those with no conditions. Furthermore, we found modular closeness between the four gene sets as well as a gene network representing the intersection of our study conditions. Through functional enrichment, we found that many of the genes in each get set and the overlapping network were related to immune system processes, thereby suggesting a functional closeness of the modules to one another and their biological interactions at early pregnancy.

Several genes in the overlapping module exhibited high centrality measures, i.e., connectivity degree with neighboring genes and betweenness centrality degree, defined as the extent to which a gene lies on the shortest path between two other genes. Genes with a high level of connectivity and betweenness centrality degrees can be leverage points in a network system owing to their control over the passage of information between network components ^27^. The genes that displayed a high level of betweenness among overlapping whole-blood gene signatures were *ARG1*, *PPBP*, and *CXCL10*. C-X-C motif chemokine 10 (*CXCL10*), a member of the chemokine family, has been previously linked in the literature to PE, with one study finding significantly higher levels of CXCL10 in PE patients versus controls^28^. Another investigation reported that vitamin D receptor agonists exert a suppression of inflammatory processes and reduce the release of CXCL10^29^. A few studies have shown an inverse relationship of serum CXCL10 and vitamin D concentrations in autoimmune diseases^29,30^ that could potentially be rectified with vitamin D supplementation^31,32^. CXCL10 is also a powerful chemokine inducing Th1-mediated responses (i.e. IFN-gamma and IL-2) whose suppressions are essential for a shift in TH1/TH2 balance for a normal pregnancy^33^; higher levels of CXCL10 have been shown in PE^34^. Relevantly, vitamin D receptor (VDR) agonists could switch the immune system cell balance from Th1 to Th2 dominance while counteracting CXCL10 production and release by different cell types^29^.

Increased expression of arginase 1 (*ARG*1), an anti-inflammatory-specific gene has been linked to allergic airway inflammation and asthma^35^ and vitamin D-mediated inflammation inhibition^36^. ARG1 is an important effector molecule for granulocytic myeloid-derived suppressor cells (G-MDSCs) which maintain maternal–fetal tolerance during pregnancy. ARG1 has found to be reduced in PE patients compared to healthy pregnant women resulting in inhibition of the development of G-MDSCs^37^. Additionally, one characteristic of hypertension, a major feature of PE, is a decreased endothelium-dependent nitric oxide (NO)-mediated vasodilation. The upregulation of vascular ARG1, which is involved in L-arginine/NO metabolism, could also contribute to decreased NO-mediated vasodilation and hypertension^38^.

Pro-Platelet Basic Protein (*PPBP*), another highly connected gene in our network, has been identified as a potential placental biomarker for PE and asthma in the literature^39,40^. Other noteworthy genes include cysteine-rich secretory protein 3 (*CRISP3*), which has also been linked to the development of PE, asthma, and weight gain^41–43^ and metalloproteinase-8 (*MMP8*) which has been reported in association with pre and in-pregnancy excess BMI, PE, and asthma as well^46,47,4445^. Notably, our peripheral blood transcriptome analysis of spontaneous preterm birth (sPTB: 24–32 wga vs Term: > 37 wga) and vitamin D insufficiency status (< 30 ng/mL) in mothers from the VDAART revealed *CRISP3*, *ARG1,* and *MMP8* as highly connected genes in the overlapping module^48^. These genes might play an important role in the pathobiology of both PE and sPTB at earlier stages of pregnancy and their function might be modified by vitamin D status during pregnancy as well as other maternal risk factors.

### Early-pregnancy biological pathways of preeclampsia in the placenta

PE is clinically defined by the secondary features of a primary placental disorder ^22,49^. 73% of human proteins are expressed in the placenta^50^. Genes expressed in the blood could overlap with 80% of genes expressed in other tissues^51^. Pregnancy is accompanied by longitudinal physiological changes that affect maternal proteome in peripheral blood^52^. Early-pregnancy characterization of the maternal proteins associated with PE is essential to understanding the pathobiology of the condition at the pre-clinical stage and may help us predict pregnancy outcomes and identify preventive measures.

Upon replicating of the peripheral blood intersecting genes from the first part of our analysis in two separate placental expression datasets and mapping these to the PPI, we identified several important patterns. In the module of overlapping gene signatures appearing in the placentae from preeclamptic pregnancies that were differentially expressed compared to the term-placental of uncomplicated pregnancies, we identified *MMP8*, *CRISP3*, and *PPBP* among the most highly connected genes (Fig. 7). As noted earlier, all three of these genes have implications in PE and at least one of the other study conditions. Similarly, we found that highly connected genes from the peripheral blood module appeared in the placentae from early to mid-pregnancies. Among these were *CXCL10*, *PPBP*, and *ARG1*. While some genes related to abnormal placental function may be trimester dependent (Fig. 6), other genes such as *CRISP3* are expressed throughout normal pregnancy and demonstrated altered expression in the process of PE development. Of note, the role of epithelial and neutrophil-derived CRISP3 has been implicated in the enhancement of innate, and adaptive immunity as well as endometrial adhesion, proliferation, and regeneration^53,54^.

Abnormal placental function and maternal trophoblastic interaction (syncytiotrophoblast layer in direct contact with maternal blood) are recognized to be central in the pathogenesis of PE^55^. Nevertheless, PE is a multifactorial disorder of pregnancy, in part due to maternal factors. In a normal pregnancy, maternal innate immune responses are altered to an enhanced inflammatory state. This state may play a beneficial role throughout pregnancy, as inflammatory cytokines are involved in the processes of implantation and placentation and may also help the mother to fight infection due to suppression of her T and NK cell-mediated immune responses^49^; however, the activation of the innate immune response is more extreme in PE which highlights the pivotal role of maternal cells in inducing the cytokine profile at the maternal–fetal interface^49,56,57^. Our study implicates the role of maternal risk factors in triggering dysregulation of normal biological processes in the placenta that might result in PE development. The early and mid-pregnancy peripheral blood gene signatures that we identified and confirmed in the placenta provided insight into the factors that trigger the onset of PE, rather than mechanisms that have already occurred. This finding sets a paradigm for future investigations using peripheral blood gene signatures to learn more about diseases that originate at early pregnancy in the placenta.

The shared gene signature we identified across four study conditions provides evidence for tissue-specific gene signatures that not only link these conditions together but might allow us to identify high-risk women predisposed to PE in early pregnancy given a sample of peripheral blood. Furthermore, this gene signature provides insight into disease conditions that are associated with the later development of PE, a finding that can help direct early intervention for the prevention of PE. The shared gene signatures between these comparisons suggest that deviation of normal placenta biological process is partly trimester-dependent which could be influenced by maternal factors (Fig. 6).

A limitation of this study was the sample size of the replication cohorts in peripheral blood and placenta from normal pregnancy which might have affected the size of the corresponding modules. Nevertheless, this limitation was inevitable based on the currently available datasets relevant to this study by pregnancy time points and conditions. Additionally, maternal asthma status was determined based on the participant’s report of physician-diagnosed asthma.

In conclusion, our investigation revealed a network that represents common gene signatures in peripheral encompassing PE, asthma, excess BMI and insufficient vitamin D status at early pregnancy. We found that these DE peripheral blood genes were also present in placental tissue, suggesting that the mechanisms in which maternal asthma, BMI, and insufficient vitamin D put women at risk for PE may come into effect during early pregnancy through affecting the immune responses or dysregulation of biological processes in the placenta. Based on these findings, further studies to distinguish the key placental intrinsic and extrinsic factors responsible for the establishment of normal pregnancy or derailment to PE are warranted.

## Methods

### Study cohorts

Figure 1 summarizes the study cohorts and our stepwise approach in this study. Supplemental File S3 provides the details of the discovery and replication cohorts as well as the assessments of PE, maternal asthma, BMI and vitamin D status. Microarray processing and quality control of gene expression data for whole blood gene expression profiling are also detailed in Supplemental File S3. The VDAART protocol for primary and ancillary studies was approved by the Institutional Review Board (IRB) at Brigham and Women’s Hospital (IRB Protocol Number: 2009P000557); all research was performed in accordance with relevant regulations and guidelines. All subjects from VDAART gave their written informed consent to participate in this ancillary study which was approved by the IRB.

### Differential gene expression analysis and overlapping gene signatures in peripheral blood

I. *Transcriptomic variations related to preeclampsia at early pregnancy*

After a surrogate variable analysis and adjustment for expression heterogeneity, we carried out differential gene expression analysis of genes related to PE at the preclinical stage (10–18 wga) using the Bioconductor “RankProd” package. The analytical package implements the Rank Product (RP) method for identifying the DE genes that are consistently upregulated (or downregulated) in a number of experiment samples. Such an approach and the applied non-parametric statistic by the RP method allow for increased performance in the case of small sample size and heterogeneity of samples^58,59^. DE genes were identified through permutation analysis, after setting the percentage of the false prediction threshold < 0.05. We identified genes associated with PE case status using the same procedure on the OMEGA cohort. At the replication step, those discovered genes associated with PE having a two-sided *P*-value < 0.05 in the replication sets were considered for determining the overlap with transcriptome signatures associated with maternal asthma, vitamin D insufficiency, and excess BMI.

II. *Transcriptomic variations related to maternal asthma, BMI and vitamin D status*

Using the same rank product method, we separately identified genes associated with vitamin D insufficiency status (< 30 ng/ml), maternal asthma status, and excess BMI (≥ 25 kg/m^2^) at 10–18 wga in the discovery VDAART group (N = 157). Similar to the PE transcriptome analysis, DE (up- or downregulated) genes were identified through permutation analysis, after setting the percentage of the false prediction threshold < 0.05. We identified DE genes associated with each pregnancy condition, i.e., maternal asthma, vitamin D status, and BMI status, using the same procedure in an extra set of pregnant women from the VDAART (N = 24) without PE. At the replication step, those discovered genes having a two-tailed *P*-value < 0.05 obtained for each condition were considered for the identification of the overlapping transcriptome signatures.

III. *Differential expression sensitivity analysis in peripheral blood*

For a sensitivity analysis, we identified the DE genes between subjects with all conditions (with PE, asthma, vitamin D < 30 ng/ml and BMI ≥ 25 kg/m^2^: N = 15) and those with none (without PE, asthma, vitamin D < 30 ng/ml and BMI ≥ 25 kg/m2: N = 13) in the VDAART cohort (*P*-value < 0.05). We determined how many of these DE genes were also present in the replicated overlapping gene signatures.

IV. *Pathway enrichment analysis and functional similarity of gene signatures*

To obtain biological knowledge of the replicated gene signatures and categorize them according to their known biological functions, we conducted Gene Ontology (GO) enrichment analysis (EA). GOEA returns the functional roles of a gene set organized by three orthogonal aspects: biological processes, molecular function, and cell component^60^. Biological process refers to the biological objective to which the gene contributes, molecular function refers to the biochemical activity of the gene product, and cellular component refers to the location in which the gene product is active^60^. Ordered functional enrichment representative of annotated GO terms for each of the 4 replicated gene sets (PE, maternal asthma, vitamin D insufficiency, and excess BMI) was separately carried out with g: Profiler^61^. Accordingly, gene group functioning profiling was performed with default options limiting the output to significant results (multiple testing-corrected *P* < 0.05). Within each of the enriched GO terms, the involved genes of each condition were listed.

To assess the pairwise functional similarity in humans for our 4 gene signatures and their overlap, we used the mclusterSim function in the R package GOSemSim to analyze the overall similarity of the lists of gene signatures with respect to their GO annotations^62^. GOSemSim integrates multiple functional similarity algorithms to compute functional similarities between gene products based on the information content of GO terms and the similarity of their associated GO annotation. To compute semantic similarity among the sets of our gene products, we used the “Wang” method that scores the semantic similarity of two GO terms based on both the locations of these terms in the GO graph and their relations with their ancestor terms^62^. The semantic similarity scores of multiple GO terms associated with gene products were combined using a “Best Match Strategy (BMA)” to a score between 0 to 1, indicating the degree of the GO term and functional similarity of compared sets of gene signatures. The closer the numeric value is to one, the greater the similarity in GO terms and functions of the two sets of genes.

V. *Direct interaction of transcriptome signatures in the interactome, the module closeness, and their overlap*

We examined the local clustering and physical interactions of the early-pregnancy replicated gene signatures for each gene set by mapping them to the human interactome (protein–protein interaction [PPI] network) using the R Bioconductor package “STRINGdb” which is the R interface for the STRING PPI database^63^. We used direct interactions between genes (proteins) with a combined confidence score > 0.4 which provides more than 50% confidence in the proposed PPIs^63^. The results were updated with the most recent STRING database, “v11.0,” available online (https://string-db.org/). We used the information from the PPI database and each of the four sets of DE genes to construct observable modules for each disease condition. We further measured the closeness of PE module to maternal asthma, BMI and vitamin D modules in comparison to unrelated conditions in the PPI (Supplemental File S3; Figs. S3A & S3B).

The intersection of the 4 sets of replicated DE gene signatures related to the study conditions (PE ∩ maternal asthma ∩ vitamin D insufficiency ∩ excess BMI gene signatures) was determined. The overlap of gene signatures was used for further investigation of their direct interactions in the interactome and largest connected component (LCC), GOEA and pathway analyses, network visualization, literature curation, as well as tissue specificity in the placenta. We curated the overlapping gene signatures in association with PE using 3 resources of GeneCards^64^, MetaCore from Clarivate Analytics^65^ and literature search (PubMed and Google Scholar).

VI. *Placental expression of peripheral blood overlapping gene signatures*

To assess the expression variations in the overlap of peripheral blood gene signatures with the placenta during the studied period (10–18 wga) as well as under pregnancies with PE, we used two Gene Expression Omnibus (GEO) datasets (https://ncbi.nlm.nih.gov/geo). GEO is an online public repository for microarray and high-throughput expression data maintained by the National Center for Biotechnology Information (NCBI)^66^. The datasets were both on the platform of Affymetrix Human Gene 1.0 ST Array.

Entrez ID was used to integrate expression data from multiple microarray platforms ^67^. The first database consisted of microarray placental gene expression profiles of 12 samples obtained from 1st and 2nd trimesters of healthy pregnancies (45–59 days and 109–115, respectively) and term, four samples for each time point (GSE9984)^68^. The raw data were downloaded, and background adjustment, log_2_ transformation, and quantile normalization on the arrays were applied by “rma” function in R BioConductor’s “affy” library. We first examined whether the overlapping gene signatures were expressed in all samples across the 3 pregnancy time points. Next, we conducted SVA-adjusted differential gene expression analysis using the RankProd method on the 1^st^ and 2^nd^-trimester samples, approximately corresponding to the gestational age of samples in our study (10–18 wga). We examined whether the overlapping gene signatures, at least in part, were also differentially expressed in the placenta of early to mid-healthy pregnancy (*P* < 0.05).

To assess whether the overlapping gene signatures were also differentially expressed in the placentae of pregnancies with PE compared to the placentae of healthy pregnancies, we used a second GEO database of human placental microarray data (GSE75010). This dataset contains term-placental gene expression profiles of 80 and 77 pregnancies with and without PE, respectively^69^. Differential gene expression analysis was conducted using the same methodology as described above to identify a subset of overlapping gene signatures differentially expressed in the placenta from pregnancies with PE compared with those from healthy pregnancies.

## Supplementary information

Supplementary information 1.

Supplementary information 2.

Supplementary information 3.

## Data Availability

The data that support the findings of this study are available in the Gene Expression Omnibus (GEO) database at: https://www.ncbi.nlm.nih.gov/geo/query/acc.cgi?token=epajoakqppkvbub&acc=GSE85307 reference number GSE85307. Additional data were derived from the following resources available in the public domain of GEO database: https://www.ncbi.nlm.nih.gov/geo/query/acc.cgi?acc=GSE9984, https://www.ncbi.nlm.nih.gov/geo/query/acc.cgi?acc=GSE75010, and https://www.ncbi.nlm.nih.gov/geo/query/acc.cgi?acc=GSE142974.
