# Supplementary material for: Early-pregnancy transcriptome signatures of preeclampsia: from peripheral blood to placenta
Source: Sci Rep. 2020 Oct 12;10:17029. doi: 10.1038/s41598-020-74100-1 (PMC7550614; doi:10.1038/s41598-020-74100-1)
Supplement: Supplementary file 1 — Supplementary information 1. [file 41598_2020_74100_MOESM1_ESM.docx]

**Early-Pregnancy Transcriptome Signatures of Preeclampsia:**

**From Peripheral Blood to Placenta**

Aishwarya P. Yadama, BS^1^; Enrico Maiorino, PhD^1^; Vincent J. Carey, PhD^1^; Thomas F. McElrath, MD, PhD^2^; Augusto A. Litonjua, MD, MPH^3^; Joseph Loscalzo, MD, PhD^4^; Scott T. Weiss, MD, MS^1^; Hooman Mirzakhani, MD, PhD, MMSc^1^

**Supplementary File 1 (S1):** Vitamin D Antenatal Asthma Reduction Trial (VDAART) Eligibility Criteria

Two subsets of enrolled pregnant women in the Vitamin D Antenatal Asthma Reduction Trial were used in this study. Accordingly, inclusion and exclusion criteria for those subjects were per trial protocol.

**Vitamin D Antenatal Asthma Reduction Trial (VDAART) Eligibility Criteria**

***Inclusion Criteria***

- Maternal personal or biological father history of asthma, eczema, allergic rhinitis. Pregnant women were considered asthmatic or allergic if they had a positive answer to the direct question in the enrollment questionnaire or any monthly subsequent questionnaire.
- Gestational age between 10 and 18 weeks at the time of randomization
- Maternal age between 18 and 39 years
- Not a current smoker (defined as not having smoked for at least 1 month prior to enrollment) and not a user of other nicotine products (e.g., nicotine patch) for at least 1 month prior to enrollment
- English- or Spanish-speaking
- Intent to participate through pregnancy

***Exclusion Criteria***

- Gestational age >18 weeks
- Presence of chronic medical conditions: (i) hypertension on medications, (ii) diabetes mellitus, *(III)* parathyroid disease, (iv) uncontrolled thyroid disease, v) kidney stones, and (vi) sarcoidosis
- Intake of vitamin D supplements containing > 2,000 IU/day of vitamin D3
- Multiple gestation pregnancy
- Pregnancy achieved by assisted reproduction techniques (e.g. IUI, IVF)
- Current use of illicit drugs (defined as any use in the past 6 months prior to enrollment)
- Previously enrolled in the VDAART for a prior pregnancy
- Any major fetal anomalies detected prior to delivery
- Patient Health Questionnaire (PHQ-9) depression scale ≥ 15
- Any condition, in the opinion of the Clinical Center Principal Investigator, which would inhibit compliance with the study medications or prohibit long-term participation in the trial.
