# Supplementary material for: Early-pregnancy transcriptome signatures of preeclampsia: from peripheral blood to placenta
Source: Sci Rep. 2020 Oct 12;10:17029. doi: 10.1038/s41598-020-74100-1 (PMC7550614; doi:10.1038/s41598-020-74100-1)
Supplement: Supplementary file 2 — Supplementary information 2. [file 41598_2020_74100_MOESM2_ESM.docx]

**Early-Pregnancy Transcriptome Signatures of Preeclampsia:**

**From Peripheral Blood to Placenta**

Aishwarya P. Yadama, BS; Vincent J. Carey, PhD; Enrico Maiorino, PhD; Thomas F. McElrath, MD, PhD; Augusto A. Litonjua, MD, MPH; Joseph Loscalzo, MD, PhD; Scott T. Weiss, MD, MS; Hooman Mirzakhani, MD, PhD, MMSc

**Supplementary File 2 (S2)**

**Supplemental Table S2A:** Overlapping gene signatures of the replicated gene signatures associated with preeclampsia, maternal asthma, BMI and vitamin D status

**Supplemental Table S2B:** Pathway enrichment analysis of the overlapping gene signatures

**Supplemental Table S2C:** Differentially expressed genes in subjects with all conditions versus subjects with none of the conditions

**Supplemental Table S2D:** Overlapping gene signatures enriched in healthy placentas (1^st^ trimester vs 2^nd^ trimester)

**Supplemental Table S2E:** Overlapping gene signatures enriched in placenta (pregnancies with preeclampsia vs healthy/uncomplicated pregnancies)

**Supplemental Table S2A**. **Overlapping gene signatures of the replicated gene signatures associated with preeclampsia, maternal asthma, BMI and vitamin D status**

|  | Gene Symbol | Probe ID | P-Value | Regulation | Fold Change | LCC Overlap | LCC Healthy Placenta Module | LCC Preeclampsia Placenta | Genecards | Metacore | Manual Annotations | Any |
| --- | --- | --- | --- | --- | --- | --- | --- | --- | --- | --- | --- | --- |
| 1 | AHSP | 7995237 | 1.92E-05 | Down | 0.98 | Yes | Yes | No | Curated | Not curated | Not reported | Yes |
| 2 | AK5 | 7902452 | 0.000259 | Down | 0.99 | Yes | No | No | Not curated | Not curated | Not reported | No |
| 3 | ALOX15 | 8011680 | 5.27E-09 | Down | 0.98 | Yes | No | No | Curated | Not curated | Not reported | Yes |
| 4 | ANKRD22 | 7934898 | 0.001235 | Up | 1.05 | Yes | Yes | No | Not curated | Not curated | Not reported | No |
| 5 | ARG1 | 8122058 | 3.01E-05 | Up | 1.023 | Yes | Yes | No | Curated | Not curated | [Wang et al. 2018](https://www.ncbi.nlm.nih.gov/pubmed/29763854) | Yes |
| 6 | ARRDC4 | 7986350 | 0.000239 | Down | 0.93 | No | No | No | Not curated | Not curated | Not reported | No |
| 7 | BANK1 | 8096617 | 3.02E-08 | Up | 1.06 | Yes | No | No | Not curated | Not curated | Not reported | No |
| 8 | BPGM | 8136341 | 0.001071 | Up | 1.01 | Yes | No | No | Not curated | Not curated | Not reported | No |
| 9 | BPI | 8062444 | 0.00044 | Up | 1.002 | Yes | No | Yes | Not curated | Not curated | [Chaiworapongsa et al. 2013](https://www.ncbi.nlm.nih.gov/pubmed/23793063) | Yes |
| 10 | C17orf97 | 8003607 | 0.001334 | Down | 0.96 | Yes | No | No | Not curated | Not curated | [Lim et al. 2012](https://www.ncbi.nlm.nih.gov/pubmed/22901903) | Yes |
| 11 | C3AR1 | 7960874 | 1.14E-11 | Up | 1.01 | Yes | Yes | Yes | Curated | Not curated | [Gormley et al. 2017](https://pubmed.ncbi.nlm.nih.gov/28347715-preeclampsia-novel-insights-from-global-rna-profiling-of-trophoblast-subpopulations/) | Yes |
| 12 | C4BPA | 7909318 | 9.31E-20 | Up | 1.083 | Yes | Yes | No | Curated | Not curated | [Joyama et al. 2001](https://www.ncbi.nlm.nih.gov/pubmed/11136195) | Yes |
| 13 | CA2 | 8147132 | 1.36E-10 | Down | 0.93 | Yes | Yes | No | Not curated | Not curated | Not reported | No |
| 14 | CASP5 | 7951385 | 4.11E-13 | Up | 1.1 | Yes | No | No | Not curated | Not curated | Not reported | No |
| 15 | CAT | 7939298 | 1.04E-07 | Down | 0.95 | Yes | No | Yes | Curated | Not curated | [Yassaee et al. 2018](https://www.ncbi.nlm.nih.gov/pubmed/30464933) | Yes |
| 16 | CC2D2B | 7929533 | 7.70E-07 | Up | 1.04 | No | No | No | Not curated | Not curated | Not reported | No |
| 17 | CCR3 | 8079383 | 2.12E-11 | Up | 1.01 | Yes | No | No | Not curated | Not curated | [Dahlstrøm et ak. 2010](https://www.ncbi.nlm.nih.gov/pubmed/20807010) | Yes |
| 18 | CD160 | 7919243 | 4.01E-09 | Down | 0.96 | Yes | No | No | Not curated | Not curated | [Wallace et al. 2015](https://www.ncbi.nlm.nih.gov/pubmed/25381387) | Yes |
| 19 | CD177 | 8029280 | 2.20E-14 | Up | 1.01 | Yes | No | Yes | Not curated | Not curated | [Hromadnikova et al. 2014](https://www.ncbi.nlm.nih.gov/pmc/articles/PMC4266496/pdf/pone.0113735.pdf) | Yes |
| 20 | CD24 | 8177222 | 6.26E-06 | Up | 1.036 | Yes | Yes | No | Not curated | Curated | [Chaiworapongsa et al. 2013](https://www.ncbi.nlm.nih.gov/pubmed/23793063) | Yes |
| 21 | CD274 | 8154233 | 0.00019 | Up | 1.02 | Yes | Yes | No | Curated | Not curated | [Tian et al. 2016](https://www.nature.com/articles/srep27683) | Yes |
| 22 | CD79A | 8029136 | 4.10E-07 | Up | 1.05 | Yes | No | No | Not curated | Not curated | Not reported | No |
| 23 | CD93 | 8065359 | 0.000326 | Down | 0.98 | Yes | Yes | No | Not curated | Not curated | Not reported | No |
| 24 | CEACAM6 | 8029098 | 0.000969 | Up | 1.01 | Yes | No | No | Not curated | Not curated | [Chaiworapongsa et al. 2013](https://www.ncbi.nlm.nih.gov/pubmed/23793063) | Yes |
| 25 | CENPK | 8112376 | 1.00E-16 | Down | 0.967 | No | No | No | Not curated | Not curated | [Garrido-Gomez et al. 2017](https://pubmed.ncbi.nlm.nih.gov/28232601-severe-pre-eclampsia-is-associated-with-alterations-in-cytotrophoblasts-of-the-smooth-chorion/) | Yes |
| 26 | CEP78 | 8156026 | 9.60E-05 | Down | 0.96 | Yes | Yes | No | Not curated | Not curated | Not reported | No |
| 27 | CFD | 8024062 | 6.19E-14 | Down | 0.99 | Yes | Yes | No | Curated | Not curated | Not reported | Yes |
| 28 | CHI3L1 | 7923547 | 2.10E-22 | Up | 1.14 | Yes | Yes | Yes | Curated | Not curated | [Gormley et al. 2017](https://pubmed.ncbi.nlm.nih.gov/28347715-preeclampsia-novel-insights-from-global-rna-profiling-of-trophoblast-subpopulations/) | Yes |
| 29 | CKS2 | 8156290 | 2.85E-09 | Up | 1.05 | No | No | No | Not curated | Not curated | [Garrido-Gomez et al. 2017](https://pubmed.ncbi.nlm.nih.gov/28232601-severe-pre-eclampsia-is-associated-with-alterations-in-cytotrophoblasts-of-the-smooth-chorion/) | Yes |
| 30 | CLC | 8036755 | 1.54E-11 | Up | 1.01 | Yes | No | Yes | Not curated | Not curated | Not reported | No |
| 31 | CLEC12A | 7953901 | 2.10E-13 | Up | 1.083 | Yes | No | No | Not curated | Not curated | [Enquobahrie et al. 2011](https://www.ncbi.nlm.nih.gov/pmc/articles/PMC3077242/) | Yes |
| 32 | CLEC12B | 7953914 | 1.37E-20 | Up | 1.07 | Yes | No | No | Not curated | Not curated | [Enquobahrie et al. 2011](https://www.ncbi.nlm.nih.gov/pmc/articles/PMC3077242/) | Yes |
| 33 | CLEC4C | 7960832 | 4.06E-07 | Up | 1.02 | Yes | No | No | Not curated | Not curated | Not reported | No |
| 34 | CLEC4D | 7953749 | 4.08E-07 | Up | 1.01 | Yes | Yes | Yes | Not curated | Not curated | Not reported | No |
| 35 | CLU | 8149927 | 8.14E-09 | Down | 0.94 | Yes | Yes | Yes | Curated | Curated | [Glotov et al. 2015](https://bmcsystbiol.biomedcentral.com/articles/10.1186/1752-0509-9-S2-S4) | Yes |
| 36 | CMBL | 8110971 | 3.40E-06 | Down | 0.99 | No | No | No | Not curated | Not curated | Not reported | No |
| 37 | CPA3 | 8083260 | 9.94E-12 | Up | 1.05 | Yes | No | Yes | Not curated | Not curated | Not reported | No |
| 38 | CPT1A | 7949971 | 0.000466 | Up | 1.01 | No | No | No | Curated | Not curated | Not reported | Yes |
| 39 | CRISP3 | 8126905 | 3.15E-05 | Down | 0.92 | Yes | No | Yes | Not curated | Not curated | [Chaiworapongsa et al. 2013](https://www.ncbi.nlm.nih.gov/pubmed/23793063) | Yes |
| 40 | CSGALNACT1 | 8149574 | 1.69E-11 | Up | 1.06 | No | No | No | Not curated | Not curated | [Luo et al. 2017](file:///C:\Users\nhaya\Documents\PE_GX_ms\Tables_Supplements\ncbi.nlm.nih.gov\pmc\articles\PMC5464566\) | Yes |
| 41 | CTSW | 7941444 | 2.45E-08 | Down | 0.94 | Yes | No | No | Not curated | Not curated | Not reported | No |
| 42 | CXCL10 | 8101126 | 3.41E-09 | Down | 0.94 | Yes | Yes | No | Curated | Curated | [Liu et al. 2015](https://doi.org/10.3892/etm.2015.2337) | Yes |
| 43 | CYBRD1 | 8046333 | 0.000616 | Up | 1.02 | Yes | No | No | Not curated | Not curated | Not reported | No |
| 44 | CYP1B1 | 8051583 | 6.28E-05 | Up | 1.01 | Yes | Yes | No | Curated | Not curated | Not reported | Yes |
| 45 | CYP27A1 | 8048432 | 2.27E-11 | Down | 0.97 | No | No | No | Curated | Not curated | [Mistry et al. 2017](file:///C:\Users\nhaya\Documents\PE_GX_ms\Tables_Supplements\ncbi.nlm.nih.gov\pmc\articles\PMC5454503\) | Yes |
| 46 | DEFA4 | 8149109 | 2.01E-07 | Up | 1.01 | Yes | No | Yes | Not curated | Not curated | [Chaiworapongsa et al. 2013](https://www.ncbi.nlm.nih.gov/pubmed/23793063) | Yes |
| 47 | DHRS12 | 7971713 | 0.000674 | Up | 1.02 | No | No | No | Not curated | Not curated | Not reported | No |
| 48 | DHRS9 | 8046124 | 3.20E-07 | Up | 1.02 | No | No | No | Not curated | Not curated | Not reported | No |
| 49 | DOCK4 | 8142345 | 9.45E-06 | Down | 0.95 | No | No | No | Not curated | Not curated | [Enquobahrie et al. 2011](https://www.ncbi.nlm.nih.gov/pmc/articles/PMC3077242/) | Yes |
| 50 | DSC2 | 8022711 | 0.000142 | Up | 1.13 | Yes | Yes | No | Not curated | Not curated | [Loset et al. 2011](https://pubmed.ncbi.nlm.nih.gov/20934677-a-transcriptional-profile-of-the-decidua-in-preeclampsia/) | Yes |
| 51 | E2F2 | 7913644 | 0.000121 | Up | 1.03 | Yes | No | No | Not curated | Not curated | Not reported | No |
| 52 | ECRP | 7973108 | 1.18E-07 | Down | 0.95 | Not Mapped | Not Mapped | Not Mapped | Not curated | Not curated | Not reported | No |
| 53 | ELOVL7 | 8112274 | 6.05E-09 | Down | 0.94 | No | No | No | Not curated | Not curated | Not reported | No |
| 54 | ENC1 | 8112615 | 0.000104 | Up | 1.07 | Yes | No | No | Not curated | Not curated | Not reported | No |
| 55 | ENKUR | 7932598 | 2.39E-10 | Down | 0.92 | No | No | No | Not curated | Not curated | Not reported | No |
| 56 | ERICH1 | 8148978 | 3.56E-07 | Down | 0.96 | No | No | No | Not curated | Not curated | [Yan et al. 2013](https://www.europeanreview.org/wp/wp-content/uploads/3083-3094.pdf) | Yes |
| 57 | F2RL1 | 8106403 | 6.55E-08 | Up | 1.06 | Yes | Yes | Yes | Curated | Not curated | [Elliot et al. 2015](https://www.sfu.ca/biology/faculty/crespi/pdfs/180-ElliotCrespi2015.pdf) | Yes |
| 58 | FAM160A1 | 8097801 | 9.13E-06 | Up | 1.01 | No | No | No | Not curated | Not curated | Not reported | No |
| 59 | FAM3B | 8068684 | 0.000672 | Up | 1.01 | No | No | No | Not curated | Not curated | [Mayne et al. 2016](https://www.futuremedicine.com/doi/10.2217/epi-2016-0103) | Yes |
| 60 | FCER1A | 7906443 | 5.78E-08 | Up | 1.04 | Yes | Yes | No | Not curated | Not curated | [Rajakumar et al. 2012](https://www.ncbi.nlm.nih.gov/pmc/articles/PMC3040985/) | Yes |
| 61 | FCER2 | 8033420 | 0.000645 | Up | 1.01 | Yes | No | No | Not curated | Not curated | Not reported | No |
| 62 | FCGR2B | 7906777 | 1.67E-08 | Up | 1.02 | Yes | Yes | No | Curated | Not curated | [Enquobahrie et al. 2008](https://www.ncbi.nlm.nih.gov/pmc/articles/PMC2702488/pdf/nihms81807.pdf) | Yes |
| 63 | FCRL1 | 7921319 | 1.34E-05 | Up | 1.02 | Yes | No | No | Not curated | Not curated | Not reported | No |
| 64 | FCRL2 | 7921298 | 0.000124 | Up | 1.04 | Yes | No | No | Not curated | Not curated | Not reported | No |
| 65 | FCRL3 | 7921275 | 4.68E-05 | Down | 0.98 | Yes | No | No | Not curated | Not curated | Not reported | No |
| 66 | FCRL5 | 7921237 | 1.71E-12 | Up | 1.08 | Yes | No | No | Not curated | Not curated | Not reported | No |
| 67 | FGFBP2 | 8099471 | 6.22E-07 | Down | 0.99 | Yes | No | No | Not curated | Not curated | Not reported | No |
| 68 | FLVCR1 | 7909628 | 0.000824 | Up | 1.03 | Yes | No | No | Not curated | Not curated | Not reported | No |
| 69 | FMN1 | 7987145 | 4.18E-11 | Down | 0.97 | Yes | No | Yes | Not curated | Not curated | Not reported | No |
| 70 | FOLR3 | 7942328 | 4.45E-10 | Up | 1.11 | Yes | No | No | Not curated | Not curated | Not reported | No |
| 71 | GATM | 7988414 | 1.58E-07 | Up | 1.06 | Yes | Yes | No | Not curated | Not curated | Not reported | No |
| 72 | GBP5 | 7917576 | 0.000539 | Up | 1.01 | Yes | Yes | No | Not curated | Not curated | [Gomez et al. 2017](https://dev.biologists.org/content/develop/early/2017/02/21/dev.146100.full.pdf) | Yes |
| 73 | GM2A | 8109344 | 3.20E-06 | Down | 0.98 | Yes | Yes | No | Not curated | Not curated | [White et al. 2013](https://www.ncbi.nlm.nih.gov/pmc/articles/PMC3741019/) | Yes |
| 74 | GNG11 | 8134257 | 3.00E-13 | Down | 0.89 | Yes | Yes | Yes | Not curated | Not curated | Not reported | No |
| 75 | GNLY | 8043236 | 5.92E-08 | Down | 0.96 | Yes | No | No | Curated | Not curated | [Qui et al. 2006](https://www.ncbi.nlm.nih.gov/pmc/articles/PMC1858626/pdf/nihms12975.pdf) | Yes |
| 76 | GPR146 | 8131067 | 0.000156 | Down | 0.96 | No | No | No | Not curated | Not curated | [Gormley et al. 2017](https://www.clinicalkey.com/#!/content/playContent/1-s2.0-S0002937817304416?returnurl=https:%2F%2Flinkinghub.elsevier.com%2Fretrieve%2Fpii%2FS0002937817304416%3Fshowall%3Dtrue&referrer=) | Yes |
| 77 | GRAMD1C | 8081758 | 6.56E-08 | Up | 1.05 | No | No | No | Not curated | Not curated | [Ren et al. 2019](https://www.biorxiv.org/content/10.1101/787796v1.full.pdf) | Yes |
| 78 | GSTM1 | 7903765 | 6.26E-39 | Up | 1.28 | Yes | Yes | No | Curated | Curated | [Guan et al. 2016](https://www.clinicalkey.com/service/content/pdf/watermarked/1-s2.0-S0301211516308181.pdf?locale=en_US&searchIndex=) | Yes |
| 79 | GSTT1 | 8074980 | 1.47E-08 | Down | 0.96 | Not Mapped | Not Mapped | Not Mapped | Curated | Curated | [Guan et al. 2016](https://www.clinicalkey.com/service/content/pdf/watermarked/1-s2.0-S0301211516308181.pdf?locale=en_US&searchIndex=) | Yes |
| 80 | GTSF1 | 7963817 | 0.000147 | Up | 1.03 | Yes | No | No | Not curated | Not curated | Not reported | No |
| 81 | GYPA | 8102998 | 1.46E-08 | Up | 1.02 | Yes | Yes | No | Not curated | Not curated | [Mousa et a. 2012](https://scholarscompass.vcu.edu/cgi/viewcontent.cgi?article=3644&context=etd) | Yes |
| 82 | GYPB | 8102993 | 4.80E-09 | Up | 1.07 | Yes | Yes | No | Not curated | Not curated | [Wilson et al. 2017](https://open.library.ubc.ca/cIRcle/collections/ubctheses/24/items/1.0361940) | Yes |
| 83 | GYPE | 8102988 | 0.000214 | Down | 0.99 | Yes | No | No | Not curated | Not curated | Not reported | No |
| 84 | GZMH | 7978360 | 5.62E-06 | Down | 0.97 | Yes | No | No | Not curated | Not curated | [Blair et al. 2013](https://open.library.ubc.ca/cIRcle/collections/ubctheses/24/items/1.0074068) | Yes |
| 85 | GZMK | 8105331 | 1.19E-06 | Down | 0.98 | Yes | No | No | Not curated | Not curated | [Liu et al. 2019](https://www.ncbi.nlm.nih.gov/pmc/articles/PMC6722495/pdf/bsr-39-bsr20190187.pdf) | Yes |
| 86 | HBM | 7991758 | 3.63E-07 | Up | 1.03 | Yes | No | No | Not curated | Not curated | Not reported | No |
| 87 | HBZ | 7991750 | 1.78E-10 | Down | 0.89 | Yes | Yes | Yes | Not curated | Not curated | Not reported | No |
| 88 | HEMGN | 8162719 | 5.91E-05 | Down | 0.96 | Yes | No | No | Not curated | Not curated | Not reported | No |
| 89 | HIP1 | 8140319 | 3.00E-07 | Down | 0.98 | No | No | No | Not curated | Not curated | Not reported | No |
| 90 | HLA-DOB | 8178833 | 0.000767 | Down | 0.98 | Yes | No | No | Not curated | Not curated | [Chu et al. 2014](https://www.ncbi.nlm.nih.gov/pmc/articles/PMC4172433/) | Yes |
| 91 | HLA-DPA1 | 8178891 | 3.54E-14 | Up | 1.12 | Yes | Yes | No | Not curated | Not curated | [Centlow et al. 2008](https://www.ncbi.nlm.nih.gov/pubmed/18166190) | Yes |
| 92 | HLA-DPB1 | 8179519 | 3.33E-09 | Up | 1.07 | Yes | Yes | No | Curated | Not curated | [Honda et al. 2000](https://www.ncbi.nlm.nih.gov/pubmed/10960630) | Yes |
| 93 | HLA-DQB1 | 8125461 | 1.10E-28 | Up | 1.05 | Yes | Yes | No | Curated | Curated | [Honda et al. 2000](https://www.ncbi.nlm.nih.gov/pubmed/10960630) | Yes |
| 94 | HORMAD1 | 7919787 | 0.000117 | Down | 0.98 | No | No | No | Not curated | Not curated | Not reported | No |
| 95 | HP | 7997188 | 6.26E-15 | Up | 1.1 | Yes | Yes | Yes | Curated | Curated | [Michita et al. 2018](https://www.frontiersin.org/articles/10.3389/fphys.2018.01771/full) | Yes |
| 96 | HRH4 | 8020684 | 9.25E-11 | Up | 1.02 | Yes | No | No | Not curated | Not curated | Not reported | No |
| 97 | IDO1 | 8146092 | 0.001007 | Up | 1.05 | Yes | Yes | Yes | Curated | Curated | [Nishizawa et al. 2010](https://www.ncbi.nlm.nih.gov/pubmed/20192952) | Yes |
| 98 | IFI27 | 7976443 | 1.26E-09 | Up | 1.04 | Yes | Yes | No | Curated | Not curated | Not reported | Yes |
| 99 | IFI44 | 7902553 | 5.80E-06 | Up | 1.01 | Yes | Yes | No | Not curated | Not curated | [Leavey et al. 2015](https://pubmed.ncbi.nlm.nih.gov/25679511-large-scale-aggregate-microarray-analysis-reveals-three-distinct-molecular-subclasses-of-human-preeclampsia/) | Yes |
| 100 | IFI44L | 7902541 | 5.15E-07 | Up | 1.02 | Yes | Yes | No | Not curated | Not curated | Not reported | No |
| 101 | IFI6 | 7914127 | 5.42E-08 | Up | 1.06 | Yes | No | No | Not curated | Not curated | [Ugolini-Lopes et al. 2019](https://pubmed.ncbi.nlm.nih.gov/30772492-enhanced-type-i-interferon-gene-signature-in-primary-antiphospholipid-syndrome-association-with-earlier-disease-onset-and-preeclampsia/) | Yes |
| 102 | IFIT1 | 7929065 | 0.002032 | Up | 1.09 | Yes | Yes | No | Not curated | Not curated | [Ren et al. 2019](https://www.biorxiv.org/content/10.1101/787796v1.full.pdf) | Yes |
| 103 | IGF2BP2 | 8092552 | 1.53E-06 | Down | 0.95 | No | No | No | Not curated | Not curated | Not reported | No |
| 104 | IGKC | 8043433 | 3.51E-13 | Up | 1.1 | Not Mapped | Not Mapped | Not Mapped | Not curated | Not curated | Not reported | No |
| 105 | IL18RAP | 8044049 | 1.00E-05 | Up | 1.1 | Yes | No | No | Not curated | Not curated | [Hu et al. 2009](https://www.ncbi.nlm.nih.gov/pubmed/19642860) | Yes |
| 106 | IL1R1 | 8043995 | 3.24E-05 | Down | 0.96 | Yes | Yes | No | Curated | Not curated | [Mousa et al. 2012](https://www.ncbi.nlm.nih.gov/pubmed/22902744) | Yes |
| 107 | IL23R | 7902189 | 0.000886 | Up | 1.04 | Yes | No | No | Not curated | Not curated | [Jahantigh et al. 2019](https://www.ncbi.nlm.nih.gov/pubmed/31123953) | Yes |
| 108 | IL5RA | 8085062 | 3.57E-10 | Up | 1.01 | Yes | Yes | No | Not curated | Not curated | [Enquobahrie et al. 2008](https://www.ncbi.nlm.nih.gov/pubmed/18533121) | Yes |
| 109 | ISCA1 | 8112331 | 6.84E-07 | Down | 0.95 | No | No | No | Not curated | Not curated | Not reported | No |
| 110 | ITGA2B | 8016044 | 3.50E-07 | Down | 0.96 | Yes | No | No | Curated | Not curated | Not reported | Yes |
| 111 | ITGB3 | 8007931 | 1.70E-12 | Down | 0.94 | Yes | No | Yes | Curated | Curated | [Calicchio et al. 2013](https://www.sciencedirect.com/science/article/pii/S0002944013006068) | Yes |
| 112 | JUP | 8015412 | 1.10E-05 | Up | 1.03 | Yes | Yes | Yes | Not curated | Not curated | [Ren et al. 2019](https://www.biorxiv.org/content/10.1101/787796v1.full.pdf) | Yes |
| 113 | KEL | 8143534 | 2.50E-05 | Up | 1.04 | Yes | No | No | Not curated | Not curated | Not reported | No |
| 114 | KIAA0825 | 8113097 | 0.001101 | Down | 0.98 | No | No | No | Not curated | Not curated | Not reported | No |
| 115 | KIAA1324 | 7903592 | 4.59E-29 | Down | 0.81 | No | No | No | Not curated | Not curated | Not reported | No |
| 116 | KIAA1324L | 8140709 | 3.54E-08 | Down | 0.96 | No | No | No | Not curated | Not curated | Not reported | No |
| 117 | KLRF1 | 7953892 | 8.51E-16 | Down | 0.92 | Yes | No | No | Not curated | Not curated | [Sun et al. 2009](https://www.ncbi.nlm.nih.gov/pubmed/19681734) | Yes |
| 118 | KLRG1 | 7953835 | 3.85E-08 | Up | 1.05 | Yes | No | No | Not curated | Not curated | Not reported | No |
| 119 | KRT23 | 8015133 | 7.44E-06 | Down | 0.98 | Yes | No | No | Not curated | Not curated | [Loset et al. 2011](https://www.ncbi.nlm.nih.gov/pmc/articles/PMC3011026/pdf/nihms234235.pdf) | Yes |
| 120 | KRT73 | 7963471 | 0.00045 | Up | 1.01 | Yes | No | No | Not curated | Not curated | Not reported | No |
| 121 | LCN2 | 8158167 | 4.10E-05 | Down | 0.97 | Yes | Yes | No | Curated | Not curated | [Chaiworapongsa et al. 2013](https://www.ncbi.nlm.nih.gov/pubmed/23793063) | Yes |
| 122 | LGALS2 | 8075956 | 1.01E-07 | Down | 0.96 | No | No | No | Not curated | Not curated | [Blois et al. 2019](https://www.frontiersin.org/articles/10.3389/fimmu.2019.01166/full) | Yes |
| 123 | LGALSL | 8042283 | 3.97E-05 | Down | 0.98 | Yes | No | No | Not curated | Not curated | [Gormley et al. 2017](https://cyberleninka.org/article/n/1494418) | Yes |
| 124 | LILRA3 | 8039226 | 2.54E-07 | Up | 1.1 | Not Mapped | Not Mapped | Not Mapped | Not curated | Not curated | [Sun et al. 2009](https://www.ncbi.nlm.nih.gov/pubmed/19681734) | Yes |
| 125 | LINC00189 | 8068100 | 8.99E-12 | Up | 1.01 | Not Mapped | Not Mapped | Not Mapped | Not curated | Not curated | Not reported | No |
| 126 | LINC00328 | 7904478 | 4.12E-07 | Up | 1.01 | Not Mapped | Not Mapped | Not Mapped | Not curated | Not curated | Not reported | No |
| 127 | LPAR1 | 8163257 | 1.37E-10 | Down | 0.9 | Yes | Yes | Yes | Not curated | Not curated | [Fujii et al. 2019](https://onlinelibrary.wiley.com/doi/abs/10.1111/aji.13176) | Yes |
| 128 | LRRC6 | 8152962 | 5.29E-13 | Up | 1.03 | No | No | No | Not curated | Not curated | Not reported | No |
| 129 | LRRN1 | 8077366 | 2.09E-10 | Up | 1.05 | No | No | No | Not curated | Not curated | Not reported | No |
| 130 | LRRN3 | 8135488 | 1.76E-08 | Up | 1.02 | No | No | No | Not curated | Not curated | Not reported | No |
| 131 | LTF | 8086607 | 0.000124 | Down | 0.98 | Yes | No | Yes | Curated | Not curated | [Enquobahrie et al. 2011](https://www.ncbi.nlm.nih.gov/pubmed/21537405) | Yes |
| 132 | MASTL | 7926821 | 0.000204 | Down | 0.98 | Yes | No | No | Not curated | Not curated | Not reported | No |
| 133 | MFSD9 | 8054356 | 0.000693 | Up | 1.01 | No | No | No | Not curated | Not curated | Not reported | No |
| 134 | MILR1 | 8009243 | 6.06E-09 | Up | 1.01 | No | No | No | Not curated | Not curated | Not reported | No |
| 135 | MIR646HG | 8063793 | 0.001833 | Down | 0.98 | Not Mapped | Not Mapped | Not Mapped | Not curated | Not curated | Not reported | No |
| 136 | MMP8 | 7951246 | 1.88E-10 | Down | 0.99 | Yes | No | Yes | Curated | Not curated | [Rahimi et al. 2018](https://www.ncbi.nlm.nih.gov/pubmed/28745526) | Yes |
| 137 | MMRN1 | 8096415 | 1.07E-15 | Down | 0.89 | Yes | Yes | No | Not curated | Not curated | [Tejera et al. 2017](https://www.ncbi.nlm.nih.gov/pubmed/28789679) | Yes |
| 138 | MPZL1 | 7907092 | 5.48E-05 | Up | 1.01 | No | No | No | Not curated | Not curated | Not reported | No |
| 139 | MPZL2 | 7952046 | 6.19E-05 | Down | 0.99 | Yes | No | No | Not curated | Not curated | Not reported | No |
| 140 | MS4A3 | 7940216 | 1.17E-06 | Up | 1.02 | Yes | No | Yes | Not curated | Not curated | [Chaiworapongsa et al. 2013](https://www.ncbi.nlm.nih.gov/pubmed/23793063) | Yes |
| 141 | MX1 | 8068713 | 1.84E-10 | Up | 1.07 | Yes | No | No | Curated | Not curated | [Ren et al. 2019](https://www.biorxiv.org/content/10.1101/787796v1.full.pdf) | Yes |
| 142 | MYBL1 | 8151101 | 5.58E-05 | Down | 0.96 | No | No | No | Not curated | Not curated | [Tan et al. 2014](https://www.ncbi.nlm.nih.gov/pubmed/24657793) | Yes |
| 143 | MYL4 | 8007921 | 5.83E-09 | Down | 0.94 | Yes | No | No | Not curated | Not curated | [Enquobahrie et al. 2011](https://www.ncbi.nlm.nih.gov/pubmed/21537405) | Yes |
| 144 | NDUFB3 | 8047372 | 0.000105 | Down | 0.97 | No | No | No | Not curated | Not curated | Not reported | No |
| 145 | NEBL | 7932453 | 1.87E-17 | Up | 1.1 | No | No | No | Not curated | Not curated | [Lian et al. 2013](https://journals.plos.org/plosone/article?id=10.1371/journal.pone.0069848) | Yes |
| 146 | NFXL1 | 8100179 | 2.95E-13 | Down | 0.99 | No | No | No | Not curated | Not curated | Not reported | No |
| 147 | NIPAL2 | 8151952 | 6.89E-12 | Down | 0.95 | No | No | No | Not curated | Not curated | [Gormley et al. 2017](https://cyberleninka.org/article/n/1494418) | Yes |
| 148 | NLRP3 | 7911178 | 1.86E-06 | Down | 0.95 | Yes | Yes | No | Curated | Not curated | [Weel et al. 2017](https://www.ncbi.nlm.nih.gov/pubmed/28915449) | Yes |
| 149 | NMRK1 | 8161839 | 2.58E-06 | Down | 0.96 | No | No | No | Not curated | Not curated | Not reported | No |
| 150 | NOD2 | 7995539 | 3.23E-19 | Down | 0.87 | Yes | Yes | No | Curated | Curated | [Rijn et al. 2008](https://www.ncbi.nlm.nih.gov/pubmed/18382655) | Yes |
| 151 | NPCDR1 | 8088468 | 8.45E-06 | Up | 1.03 | Not Mapped | Not Mapped | Not Mapped | Not curated | Not curated | Not reported | No |
| 152 | NQO2 | 8116610 | 1.99E-05 | Down | 0.98 | No | No | No | Not curated | Not curated | [Mousa et al. 2012](https://www.ncbi.nlm.nih.gov/pubmed/22902744) | Yes |
| 153 | NRG1 | 8145766 | 7.96E-11 | Down | 0.93 | Yes | No | No | Not curated | Not curated | Not reported | No |
| 154 | NT5E | 8120967 | 0.000686 | Down | 0.99 | Yes | Yes | Yes | Curated | Not curated | [Ren et al. 2019](https://www.biorxiv.org/content/10.1101/787796v1.full.pdf) | Yes |
| 155 | ODC1 | 8050240 | 0.00056 | Down | 0.99 | Yes | No | Yes | Not curated | Not curated | [Huang et al. 2019](http://www.ijcem.com/files/ijcem0093802.pdf) | Yes |
| 156 | OLFM4 | 7969288 | 3.99E-09 | Up | 1.05 | Yes | Yes | Yes | Not curated | Not curated | [Chaiworapongsa et al. 2013](https://www.ncbi.nlm.nih.gov/pubmed/23793063) | Yes |
| 157 | OMG | 8014057 | 2.65E-08 | Up | 1.06 | No | No | No | Not curated | Not curated | Not reported | No |
| 158 | OR52K3P | 7937940 | 0.000181 | Up | 1.01 | Not Mapped | Not Mapped | Not Mapped | Not curated | Not curated | Not reported | No |
| 159 | ORAI2 | 8135172 | 4.60E-05 | Up | 1.02 | No | No | No | Not curated | Not curated | Not reported | No |
| 160 | ORM1 | 8157446 | 1.30E-35 | Up | 1.3 | Yes | No | No | Curated | Not curated | Not reported | Yes |
| 161 | OSBPL10 | 8085984 | 9.93E-07 | Up | 1.06 | No | No | No | Not curated | Not curated | Not reported | No |
| 162 | P2RX1 | 8011499 | 6.36E-05 | Down | 0.98 | Yes | No | No | Not curated | Not curated | Not reported | No |
| 163 | P2RY12 | 8091530 | 1.30E-08 | Down | 0.94 | Yes | Yes | Yes | Not curated | Not curated | [Huang et al. 2019](http://www.ijcem.com/files/ijcem0093802.pdf) | Yes |
| 164 | PADI2 | 7912937 | 3.06E-08 | Down | 0.98 | Yes | No | No | Not curated | Not curated | Not reported | No |
| 165 | PAM | 8107133 | 0.000124 | Up | 1.05 | No | No | No | Not curated | Not curated | [Ren et al. 2019](https://www.biorxiv.org/content/10.1101/787796v1.full.pdf) | Yes |
| 166 | PAPSS1 | 8102214 | 4.86E-11 | Up | 1.04 | Yes | Yes | No | Not curated | Not curated | Not reported | No |
| 167 | PAX8-AS1 | 8044605 | 4.09E-08 | Down | 0.99 | Not Mapped | Not Mapped | Not Mapped | Not curated | Not curated | [Benny et al. 2019](http://ncbi.nlm.nih.gov/pubmed/31557190) | Yes |
| 168 | PDK4 | 8141094 | 5.26E-14 | Up | 1.04 | No | No | No | Not curated | Not curated | [Yong et al. 2015](https://www.ncbi.nlm.nih.gov/pubmed/26010865) | Yes |
| 169 | PDZK1IP1 | 7915910 | 1.80E-12 | Up | 1.1 | Yes | Yes | No | Not curated | Not curated | [Leavey et al. 2018](https://www.ncbi.nlm.nih.gov/pubmed/29507646) | Yes |
| 170 | PF4V1 | 8095694 | 0.000825 | Down | 0.98 | Yes | No | No | Not curated | Not curated | [Mousa et al. 2012](https://scholarscompass.vcu.edu/cgi/viewcontent.cgi?article=3644&context=etd) | Yes |
| 171 | PITHD1 | 7898894 | 0.000164 | Down | 0.99 | No | No | No | Not curated | Not curated | Not reported | No |
| 172 | PLA2G7 | 8126784 | 5.11E-12 | Down | 0.94 | Yes | Yes | No | Curated | Not curated | [Loset et al. 2011](https://www.ncbi.nlm.nih.gov/pmc/articles/PMC3011026/) | Yes |
| 173 | PLB1 | 8041061 | 2.55E-07 | Up | 1.05 | Yes | No | No | Not curated | Not curated | Not reported | No |
| 174 | PLEK2 | 7979710 | 0.000416 | Down | 0.98 | No | No | No | Not curated | Not curated | Not reported | No |
| 175 | PPBP | 8100971 | 1.55E-12 | Down | 0.9 | Yes | Yes | Yes | Curated | Not curated | [Liu et al. 2013](https://www.ncbi.nlm.nih.gov/pubmed/24195779) | Yes |
| 176 | PRKAR2B | 8135378 | 1.88E-14 | Down | 0.88 | Yes | Yes | No | Not curated | Not curated | [Kim et al. 2016](https://www.ncbi.nlm.nih.gov/pubmed/27218821) | Yes |
| 177 | PRRG4 | 7939150 | 3.24E-07 | Down | 0.95 | Yes | No | Yes | Not curated | Not curated | Not reported | No |
| 178 | PTGS1 | 8157650 | 1.11E-06 | Down | 0.96 | Yes | Yes | No | Curated | Curated | Not reported | Yes |
| 179 | PTPRM | 8019988 | 5.02E-05 | Down | 0.97 | No | No | No | Not curated | Not curated | [Enquobahrie et al. 2011](https://www.ncbi.nlm.nih.gov/pubmed/21537405) | Yes |
| 180 | PVALB | 8075838 | 3.08E-09 | Up | 1.05 | Yes | No | No | Not curated | Not curated | Not reported | No |
| 181 | RAP1GAP | 7913385 | 2.27E-14 | Down | 0.94 | Yes | No | Yes | Not curated | Not curated | [Song et al. 2015](https://pubmed.ncbi.nlm.nih.gov/25717061-identification-of-early-onset-preeclampsia-related-genes-and-micrornas-by-bioinformatics-approaches/) | Yes |
| 182 | RBMS2 | 7956261 | 3.47E-05 | Down | 0.95 | No | No | No | Not curated | Not curated | Not reported | No |
| 183 | RHD | 7898998 | 7.51E-11 | Down | 0.95 | Yes | Yes | No | Not curated | Not curated | [Cotter et al. 2005](https://www.ncbi.nlm.nih.gov/pubmed/15842281) | Yes |
| 184 | RNASE2 | 7973110 | 2.22E-13 | Up | 1.06 | Yes | No | Yes | Not curated | Not curated | [Garrido-Gomez et al. 2017](https://www.ncbi.nlm.nih.gov/pubmed/28923940) | Yes |
| 185 | RNASE3 | 7973105 | 2.36E-13 | Down | 0.96 | Yes | No | No | Not curated | Not curated | [Chaiworapongsa et al. 2013](https://www.ncbi.nlm.nih.gov/pubmed/23793063) | Yes |
| 186 | RNASET2 | 8130768 | 0.000155 | Down | 0.96 | Yes | No | No | Not curated | Not curated | Not reported | No |
| 187 | RNF182 | 8116980 | 3.31E-30 | Up | 1.08 | No | No | No | Not curated | Not curated | [Sun et al. 2009](https://www.ncbi.nlm.nih.gov/pubmed/19681734) | Yes |
| 188 | RPL7 | 8109222 | 0.000289 | Down | 0.97 | No | No | No | Not curated | Not curated | [Yang et al. 2019](https://www.hindawi.com/journals/bmri/2019/5437621/) | Yes |
| 189 | RPS23 | 8112961 | 6.48E-09 | Down | 0.93 | No | No | No | Not curated | Not curated | Not reported | No |
| 190 | RSAD2 | 8040080 | 5.25E-08 | Up | 1.04 | Yes | Yes | No | Not curated | Not curated | [Leavey et al. 2015](https://www.ncbi.nlm.nih.gov/pubmed/25679511) | Yes |
| 191 | RUNDC3A | 8007607 | 6.50E-18 | Up | 1.13 | No | No | No | Not curated | Not curated | Not reported | No |
| 192 | S100B | 8071036 | 1.20E-14 | Down | 0.99 | Yes | No | No | Curated | Curated | [Bergman et al. 2014](https://www.ncbi.nlm.nih.gov/pubmed/24610883) | Yes |
| 193 | S100P | 8093950 | 2.00E-14 | Down | 0.99 | Yes | No | No | Not curated | Not curated | [Mousa et al. 2012](https://scholarscompass.vcu.edu/cgi/viewcontent.cgi?article=3644&context=etd) | Yes |
| 194 | SAMD12 | 8152506 | 0.000166 | Up | 1.01 | No | No | No | Not curated | Not curated | [Mousa et al. 2012](https://scholarscompass.vcu.edu/cgi/viewcontent.cgi?article=3644&context=etd) | Yes |
| 195 | SELP | 7922200 | 1.55E-07 | Down | 0.95 | Yes | Yes | Yes | Curated | Curated | [Fong et al. 2014](https://www.ncbi.nlm.nih.gov/pubmed/25028703) | Yes |
| 196 | SERPINB10 | 8021645 | 1.81E-15 | Down | 0.9 | No | No | No | Not curated | Not curated | Not reported | No |
| 197 | SERPINB9P1 | 8123606 | 0.001391 | Up | 1.01 | Not Mapped | Not Mapped | Not Mapped | Not curated | Not curated | [Sarker et al. 2014](https://www.ncbi.nlm.nih.gov/pubmed/25104112) | Yes |
| 198 | SERPING1 | 7940028 | 1.50E-07 | Up | 1.06 | Yes | Yes | Yes | Not curated | Not curated | [Low et al. 2013](https://www.ncbi.nlm.nih.gov/pubmed/24296084) | Yes |
| 199 | SH2D1B | 7921900 | 7.64E-06 | Down | 0.95 | Yes | No | No | Not curated | Not curated | [Sun et al. 2009](https://www.ncbi.nlm.nih.gov/pubmed/19681734) | Yes |
| 200 | SH3BGRL2 | 8120833 | 8.15E-11 | Down | 0.93 | No | No | No | Not curated | Not curated | [Hromadnikova et al. 2014](https://www.ncbi.nlm.nih.gov/pubmed/25502889) | Yes |
| 201 | SHISA4 | 7908758 | 0.000291 | Down | 0.96 | No | No | No | Not curated | Not curated | Not reported | No |
| 202 | SIGLEC10 | 8038824 | 0.000163 | Up | 1.03 | Yes | No | No | Not curated | Not curated | [Chaiworapongsa et al. 2013](https://www.ncbi.nlm.nih.gov/pubmed/23793063) | Yes |
| 203 | SLC12A7 | 8110755 | 4.65E-05 | Up | 1.02 | No | No | No | Not curated | Not curated | [Enquobahrie et. al 2011](https://pubmed.ncbi.nlm.nih.gov/21537405-maternal-peripheral-blood-gene-expression-in-early-pregnancy-and-preeclampsia/) | Yes |
| 204 | SLC14A1 | 8021081 | 1.84E-12 | Down | 0.92 | Yes | No | No | Not curated | Not curated | [Mousa et al. 2012](https://scholarscompass.vcu.edu/cgi/viewcontent.cgi?article=3644&context=etd) | Yes |
| 205 | SLC38A11 | 8056363 | 1.15E-05 | Up | 1.02 | No | No | No | Not curated | Not curated | Not reported | No |
| 206 | SLPI | 8066493 | 3.17E-11 | Down | 0.98 | Yes | Yes | Yes | Not curated | Not curated | [Mousa et al. 2012](https://scholarscompass.vcu.edu/cgi/viewcontent.cgi?article=3644&context=etd) | Yes |
| 207 | SMOX | 8060745 | 0.001621 | Down | 0.99 | Yes | Yes | No | Not curated | Not curated | [Lee et al. 2010](https://www.ncbi.nlm.nih.gov/pubmed/19787364) | Yes |
| 208 | SNORA68 | 8026875 | 0.000474 | Down | 0.98 | Not Mapped | Not Mapped | Not Mapped | Not curated | Not curated | Not reported | No |
| 209 | SPARC | 8115327 | 1.07E-07 | Down | 0.97 | Yes | No | No | Not curated | Not curated | [Tejera et al. 2017](https://www.ncbi.nlm.nih.gov/pubmed/28789679) | Yes |
| 210 | ST6GALNAC4 | 8164314 | 3.84E-05 | Up | 1.03 | Yes | No | Yes | Not curated | Not curated | Not reported | No |
| 211 | TARP | 8139125 | 1.33E-06 | Down | 0.98 | Not Mapped | Not Mapped | Not Mapped | Not curated | Not curated | Not reported | No |
| 212 | TCL1A | 7981183 | 1.52E-08 | Up | 1.01 | Yes | No | No | Not curated | Not curated | Not reported | No |
| 213 | TCN1 | 7948444 | 5.62E-05 | Down | 0.99 | Yes | No | No | Not curated | Not curated | [Chaiworapongsa et al. 2013](https://www.ncbi.nlm.nih.gov/pubmed/23793063) | Yes |
| 214 | THBD | 8065353 | 0.000127 | Down | 0.99 | Yes | Yes | Yes | Curated | Curated | [Chaiworapongsa et al. 2013](https://www.ncbi.nlm.nih.gov/pubmed/23793063) | Yes |
| 215 | THBS1 | 7982597 | 6.00E-10 | Down | 0.95 | Yes | Yes | Yes | Curated | Not curated | [Wang et al. 2016](https://www.ncbi.nlm.nih.gov/pubmed/27780539) | Yes |
| 216 | TMEM144 | 8098041 | 3.73E-07 | Down | 0.96 | No | No | No | Not curated | Not curated | [Triche et al. 2014](https://www.ncbi.nlm.nih.gov/pmc/articles/PMC4409136/#SD1) | Yes |
| 217 | TMEM176A | 8137264 | 5.28E-20 | Up | 1.03 | Yes | No | Yes | Not curated | Not curated | [Ren et al. 2019](https://www.biorxiv.org/content/10.1101/787796v1.full.pdf) | Yes |
| 218 | TMEM176B | 8143790 | 2.39E-13 | Up | 1.01 | Yes | No | Yes | Not curated | Not curated | [Enquobahrie et al. 2011](https://www.ncbi.nlm.nih.gov/pubmed/21537405) | Yes |
| 219 | TMTC1 | 7962058 | 4.01E-24 | Down | 0.91 | No | No | No | Not curated | Not curated | [Kaartokallio et al. 2016](https://www.ncbi.nlm.nih.gov/pmc/articles/PMC4935848/) | Yes |
| 220 | TNFAIP6 | 8045688 | 8.29E-09 | Down | 0.98 | Yes | Yes | Yes | Not curated | Not curated | [Moses et al. 2006](https://academic.oup.com/molehr/article/12/8/505/1012108) | Yes |
| 221 | TPTEP1 | 8071063 | 2.26E-10 | Down | 0.95 | Not Mapped | Not Mapped | Not Mapped | Not curated | Not curated | Not reported | No |
| 222 | TREML1 | 8126269 | 8.50E-09 | Down | 0.94 | Yes | No | No | Not curated | Not curated | Not reported | No |
| 223 | TREML3P | 8126296 | 7.13E-08 | Down | 0.97 | Not Mapped | Not Mapped | Not Mapped | Not curated | Not curated | Not reported | No |
| 224 | TREML4 | 8119427 | 1.04E-15 | Down | 0.99 | Yes | No | No | Not curated | Not curated | [Sarker et al. 2014](https://www.ncbi.nlm.nih.gov/pmc/articles/PMC4283151/) | Yes |
| 225 | TUBB2A | 8123644 | 9.32E-28 | Down | 0.82 | Yes | No | No | Not curated | Not curated | [Chang et al. 2011](https://pubmed.ncbi.nlm.nih.gov/22030040-analyses-of-placental-gene-expression-in-pregnancy-related-hypertensive-disorders/) | Yes |
| 226 | UBE2O | 8018708 | 7.66E-06 | Down | 0.97 | No | No | No | Not curated | Not curated | Not reported | No |
| 227 | USP53 | 8097098 | 0.000304 | Up | 1.02 | No | No | No | Not curated | Not curated | [Zadora et al. 2017](https://pubmed.ncbi.nlm.nih.gov/28904069-disturbed-placental-imprinting-in-preeclampsia-leads-to-altered-expression-of-dlx5-a-human-specific-early-trophoblast-marker/) | Yes |
| 228 | UTS2 | 7912136 | 7.59E-14 | Down | 0.9 | Yes | No | No | Curated | Curated | [El-Sherbiny et al. 2013](https://www.tandfonline.com/doi/full/10.3109/10641955.2013.806539) | Yes |
| 229 | VNN1 | 8129618 | 3.84E-19 | Up | 1.12 | Yes | Yes | Yes | Not curated | Not curated | Not reported | No |
| 230 | VNN3 | 8129627 | 0.000178 | Up | 1.01 | Yes | Yes | Yes | Not curated | Not curated | [Mousa et al. 2012](https://pubmed.ncbi.nlm.nih.gov/22902744-dna-methylation-is-altered-in-maternal-blood-vessels-of-women-with-preeclampsia/) | Yes |
| 231 | VSTM1 | 8039109 | 4.69E-09 | Up | 1.01 | No | No | No | Not curated | Not curated | Not reported | No |
| 232 | WLS | 7916862 | 1.61E-12 | Down | 0.95 | Yes | Yes | No | Not curated | Not curated | Not reported | No |
| 233 | XCL1 | 7907156 | 6.70E-09 | Up | 1.02 | Yes | Yes | Yes | Not curated | Not curated | Not reported | No |
| 234 | YOD1 | 7909283 | 2.76E-10 | Down | 0.96 | No | No | No | Not curated | Not curated | [Shi et al. 2015](http://europepmc.org/article/PMC/4609581) | Yes |
| 235 | ZDHHC2 | 8144758 | 3.85E-07 | Up | 1.06 | No | No | No | Not curated | Not curated | [Garrido-Gomez et al. 2017](https://dev.biologists.org/content/develop/early/2017/02/21/dev.146100.full.pdf) | Yes |
| 236 | ZNF107 | 8133049 | 0.001713 | Down | 0.99 | No | No | No | Not curated | Not curated | Not reported | No |
| 237 | ZNF641 | 7962794 | 0.000155 | Down | 0.97 | No | No | No | Not curated | Not curated | Not reported | No |
| 238 | ZNF763 | 8025978 | 0.000519 | Up | 1.02 | Not Mapped | Not Mapped | Not Mapped | Not curated | Not curated | Not reported | No |

**Supplemental Table S2B. Pathway enrichment analysis of the overlapping gene signatures**

| **GO Term Name** | **Enriched Term ID** | **Ontology Domain** | **Number of genes enriched in each term** | **Corrected P-Value for Enrichment** |
| --- | --- | --- | --- | --- |
| immune receptor activity | GO:0140375 | Molecular Function | 11 | 0.000108 |
| carbohydrate binding | GO:0030246 | Molecular Function | 15 | 0.000225 |
| tetrapyrrole binding | GO:0046906 | Molecular Function | 9 | 0.0202 |
| leukocyte mediated immunity | GO:0002443 | Biological Process | 55 | 3.44E-22 |
| immune system process | GO:0002376 | Biological Process | 103 | 6.55E-22 |
| immune response | GO:0006955 | Biological Process | 85 | 5.45E-21 |
| cell activation | GO:0001775 | Biological Process | 68 | 5.52E-21 |
| immune effector process | GO:0002252 | Biological Process | 62 | 1.27E-19 |
| regulated exocytosis | GO:0045055 | Biological Process | 48 | 2.90E-18 |
| neutrophil activation | GO:0042119 | Biological Process | 39 | 3.90E-18 |
| granulocyte activation | GO:0036230 | Biological Process | 39 | 6.43E-18 |
| leukocyte activation | GO:0045321 | Biological Process | 60 | 1.03E-17 |
| leukocyte degranulation | GO:0043299 | Biological Process | 39 | 4.11E-17 |
| myeloid leukocyte mediated immunity | GO:0002444 | Biological Process | 39 | 1.25E-16 |
| myeloid leukocyte activation | GO:0002274 | Biological Process | 42 | 1.98E-16 |
| neutrophil mediated immunity | GO:0002446 | Biological Process | 37 | 2.81E-16 |
| myeloid cell activation involved in immune response | GO:0002275 | Biological Process | 38 | 6.24E-16 |
| exocytosis | GO:0006887 | Biological Process | 48 | 7.73E-16 |
| neutrophil degranulation | GO:0043312 | Biological Process | 36 | 8.94E-16 |
| neutrophil activation involved in immune response | GO:0002283 | Biological Process | 36 | 1.09E-15 |
| leukocyte activation involved in immune response | GO:0002366 | Biological Process | 42 | 2.53E-15 |
| cell activation involved in immune response | GO:0002263 | Biological Process | 42 | 3.12E-15 |
| defense response | GO:0006952 | Biological Process | 66 | 6.29E-15 |
| secretion by cell | GO:0032940 | Biological Process | 60 | 7.53E-14 |
| secretion | GO:0046903 | Biological Process | 62 | 2.54E-13 |
| export from cell | GO:0140352 | Biological Process | 60 | 3.20E-13 |
| response to external stimulus | GO:0009605 | Biological Process | 82 | 2.05E-12 |
| response to other organism | GO:0051707 | Biological Process | 57 | 3.69E-12 |
| response to external biotic stimulus | GO:0043207 | Biological Process | 57 | 3.90E-12 |
| response to biotic stimulus | GO:0009607 | Biological Process | 57 | 9.03E-12 |
| defense response to other organism | GO:0098542 | Biological Process | 48 | 6.94E-11 |
| regulation of immune system process | GO:0002682 | Biological Process | 57 | 1.44E-10 |
| innate immune response | GO:0045087 | Biological Process | 41 | 3.41E-09 |
| inflammatory response | GO:0006954 | Biological Process | 34 | 5.44E-08 |
| positive regulation of immune system process | GO:0002684 | Biological Process | 42 | 1.96E-07 |
| regulation of leukocyte mediated immunity | GO:0002703 | Biological Process | 17 | 2.74E-07 |
| multi-organism process | GO:0051704 | Biological Process | 72 | 1.68E-06 |
| regulation of lymphocyte mediated immunity | GO:0002706 | Biological Process | 14 | 2.98E-06 |
| regulation of immune response | GO:0050776 | Biological Process | 38 | 4.01E-06 |
| response to bacterium | GO:0009617 | Biological Process | 30 | 4.5E-06 |
| positive regulation of leukocyte mediated immunity | GO:0002705 | Biological Process | 13 | 5.65E-06 |
| platelet degranulation | GO:0002576 | Biological Process | 13 | 5.65E-06 |
| response to stress | GO:0006950 | Biological Process | 86 | 7.68E-06 |
| regulation of adaptive immune response | GO:0002819 | Biological Process | 14 | 1.09E-05 |
| regulation of immune effector process | GO:0002697 | Biological Process | 22 | 3.27E-05 |
| regulation of cell activation | GO:0050865 | Biological Process | 26 | 6.55E-05 |
| regulation of response to external stimulus | GO:0032101 | Biological Process | 36 | 6.7E-05 |
| cytokine production | GO:0001816 | Biological Process | 30 | 8.37E-05 |
| vesicle-mediated transport | GO:0016192 | Biological Process | 53 | 0.000127 |
| response to molecule of bacterial origin | GO:0002237 | Biological Process | 19 | 0.000131 |
| regulation of cytokine production | GO:0001817 | Biological Process | 28 | 0.000158 |
| regulation of adaptive immune response based on somatic recombination of immune receptors built from immunoglobulin superfamily domains | GO:0002822 | Biological Process | 12 | 0.000259 |
| regulation of leukocyte activation | GO:0002694 | Biological Process | 24 | 0.000283 |
| CD4-positive, alpha-beta T cell cytokine production | GO:0035743 | Biological Process | 6 | 0.000318 |
| positive regulation of immune response | GO:0050778 | Biological Process | 30 | 0.000336 |
| regulation of defense response | GO:0031347 | Biological Process | 28 | 0.000487 |
| antimicrobial humoral response | GO:0019730 | Biological Process | 11 | 0.000506 |
| negative regulation of immune system process | GO:0002683 | Biological Process | 21 | 0.000667 |
| adaptive immune response | GO:0002250 | Biological Process | 25 | 0.000731 |
| interleukin-10 production | GO:0032613 | Biological Process | 8 | 0.000734 |
| cytokine production involved in immune response | GO:0002367 | Biological Process | 10 | 0.000866 |
| cell killing | GO:0001906 | Biological Process | 12 | 0.000998 |
| T cell cytokine production | GO:0002369 | Biological Process | 7 | 0.001072 |
| response to lipopolysaccharide | GO:0032496 | Biological Process | 17 | 0.001445 |
| regulation of cytokine production involved in immune response | GO:0002718 | Biological Process | 9 | 0.001494 |
| regulation of cell killing | GO:0031341 | Biological Process | 9 | 0.00165 |
| positive regulation of type 2 immune response | GO:0002830 | Biological Process | 5 | 0.001725 |
| cell-cell adhesion | GO:0098609 | Biological Process | 28 | 0.002001 |
| positive regulation of immune effector process | GO:0002699 | Biological Process | 13 | 0.002035 |
| leukocyte cell-cell adhesion | GO:0007159 | Biological Process | 17 | 0.002058 |
| regulation of production of molecular mediator of immune response | GO:0002700 | Biological Process | 11 | 0.002255 |
| regulation of T cell mediated immunity | GO:0002709 | Biological Process | 8 | 0.002283 |
| regulation of leukocyte mediated cytotoxicity | GO:0001910 | Biological Process | 8 | 0.002283 |
| lymphocyte mediated immunity | GO:0002449 | Biological Process | 17 | 0.00231 |
| humoral immune response | GO:0006959 | Biological Process | 17 | 0.002689 |
| regulation of type 2 immune response | GO:0002828 | Biological Process | 6 | 0.002871 |
| positive regulation of cell activation | GO:0050867 | Biological Process | 18 | 0.003381 |
| regulation of T cell cytokine production | GO:0002724 | Biological Process | 6 | 0.0035 |
| positive regulation of response to external stimulus | GO:0032103 | Biological Process | 23 | 0.003552 |
| transport | GO:0006810 | Biological Process | 94 | 0.003809 |
| T cell mediated immunity | GO:0002456 | Biological Process | 9 | 0.00416 |
| positive regulation of lymphocyte mediated immunity | GO:0002708 | Biological Process | 9 | 0.004535 |
| response to virus | GO:0009615 | Biological Process | 16 | 0.004682 |
| positive regulation of cell adhesion | GO:0045785 | Biological Process | 18 | 0.004911 |
| positive regulation of response to stimulus | GO:0048584 | Biological Process | 55 | 0.005001 |
| defense response to bacterium | GO:0042742 | Biological Process | 16 | 0.005453 |
| positive regulation of cytokine production | GO:0001819 | Biological Process | 19 | 0.005593 |
| negative regulation of interleukin-10 production | GO:0032693 | Biological Process | 5 | 0.005895 |
| regulation of interleukin-10 production | GO:0032653 | Biological Process | 7 | 0.007095 |
| regulation of antigen processing and presentation | GO:0002577 | Biological Process | 5 | 0.007664 |
| leukocyte proliferation | GO:0070661 | Biological Process | 15 | 0.0078 |
| positive regulation of cytokine production involved in immune response | GO:0002720 | Biological Process | 7 | 0.008029 |
| positive regulation of cell killing | GO:0031343 | Biological Process | 7 | 0.008029 |
| positive regulation of cell-cell adhesion | GO:0022409 | Biological Process | 14 | 0.008521 |
| positive regulation of leukocyte activation | GO:0002696 | Biological Process | 17 | 0.008709 |
| biological adhesion | GO:0022610 | Biological Process | 37 | 0.008963 |
| positive regulation of leukocyte migration | GO:0002687 | Biological Process | 10 | 0.009337 |
| type 2 immune response | GO:0042092 | Biological Process | 6 | 0.010062 |
| negative regulation of cytokine production | GO:0001818 | Biological Process | 15 | 0.01063 |
| establishment of localization | GO:0051234 | Biological Process | 94 | 0.01107 |
| regulation of T-helper 2 cell cytokine production | GO:2000551 | Biological Process | 4 | 0.011906 |
| regulation of leukocyte cell-cell adhesion | GO:1903037 | Biological Process | 15 | 0.012363 |
| response to lipid | GO:0033993 | Biological Process | 28 | 0.013396 |
| interferon-gamma production | GO:0032609 | Biological Process | 9 | 0.01481 |
| regulation of activated T cell proliferation | GO:0046006 | Biological Process | 6 | 0.015919 |
| regulation of T cell activation | GO:0050863 | Biological Process | 15 | 0.016602 |
| regulation of multicellular organismal process | GO:0051239 | Biological Process | 65 | 0.0199 |
| cell adhesion | GO:0007155 | Biological Process | 36 | 0.020416 |
| regulation of response to biotic stimulus | GO:0002831 | Biological Process | 19 | 0.02076 |
| defense response to Gram-negative bacterium | GO:0050829 | Biological Process | 8 | 0.021594 |
| regulation of cell-cell adhesion | GO:0022407 | Biological Process | 17 | 0.022761 |
| positive regulation of leukocyte mediated cytotoxicity | GO:0001912 | Biological Process | 6 | 0.024278 |
| regulation of cell adhesion | GO:0030155 | Biological Process | 23 | 0.025296 |
| T cell activation | GO:0042110 | Biological Process | 18 | 0.027316 |
| negative regulation of adaptive immune response | GO:0002820 | Biological Process | 6 | 0.027744 |
| cellular response to molecule of bacterial origin | GO:0071219 | Biological Process | 12 | 0.031293 |
| activated T cell proliferation | GO:0050798 | Biological Process | 6 | 0.031598 |
| negative regulation of dendritic cell differentiation | GO:2001199 | Biological Process | 3 | 0.033016 |
| T-helper 2 cell cytokine production | GO:0035745 | Biological Process | 4 | 0.035137 |
| positive regulation of leukocyte cell-cell adhesion | GO:1903039 | Biological Process | 12 | 0.035678 |
| positive regulation of adaptive immune response based on somatic recombination of immune receptors built from immunoglobulin superfamily domains | GO:0002824 | Biological Process | 8 | 0.037562 |
| regulation of leukocyte proliferation | GO:0070663 | Biological Process | 12 | 0.042353 |
| cytokine-mediated signaling pathway | GO:0019221 | Biological Process | 24 | 0.043432 |
| leukocyte migration | GO:0050900 | Biological Process | 18 | 0.047212 |
| adaptive immune response based on somatic recombination of immune receptors built from immunoglobulin superfamily domains | GO:0002460 | Biological Process | 15 | 0.049462 |
| secretory granule | GO:0030141 | Cell Component | 49 | 2.92E-19 |
| secretory granule lumen | GO:0034774 | Cell Component | 30 | 1.39E-16 |
| cytoplasmic vesicle lumen | GO:0060205 | Cell Component | 30 | 1.98E-16 |
| vesicle lumen | GO:0031983 | Cell Component | 30 | 2.36E-16 |
| secretory vesicle | GO:0099503 | Cell Component | 49 | 6.87E-16 |
| specific granule | GO:0042581 | Cell Component | 22 | 2.24E-15 |
| specific granule lumen | GO:0035580 | Cell Component | 15 | 6.60E-14 |
| cytoplasmic vesicle | GO:0031410 | Cell Component | 67 | 1.27E-10 |
| intracellular vesicle | GO:0097708 | Cell Component | 67 | 1.35E-10 |
| cell surface | GO:0009986 | Cell Component | 39 | 2.63E-10 |
| tertiary granule | GO:0070820 | Cell Component | 16 | 2.01E-08 |
| vesicle | GO:0031982 | Cell Component | 84 | 1.23E-07 |
| platelet alpha granule | GO:0031091 | Cell Component | 12 | 1.56E-07 |
| tertiary granule lumen | GO:1904724 | Cell Component | 10 | 2.01E-07 |
| side of membrane | GO:0098552 | Cell Component | 25 | 8.67E-06 |
| extracellular region | GO:0005576 | Cell Component | 87 | 1.72E-05 |
| extracellular space | GO:0005615 | Cell Component | 72 | 4.8E-05 |
| secretory granule membrane | GO:0030667 | Cell Component | 16 | 0.000122 |
| endomembrane system | GO:0012505 | Cell Component | 84 | 0.000161 |
| platelet alpha granule lumen | GO:0031093 | Cell Component | 8 | 0.000297 |
| external side of plasma membrane | GO:0009897 | Cell Component | 18 | 0.000336 |
| azurophil granule | GO:0042582 | Cell Component | 11 | 0.000565 |
| primary lysosome | GO:0005766 | Cell Component | 11 | 0.000565 |
| intrinsic component of membrane | GO:0031224 | Cell Component | 95 | 0.007157 |
| MHC class II protein complex | GO:0042613 | Cell Component | 4 | 0.010117 |
| cell periphery | GO:0071944 | Cell Component | 93 | 0.010579 |
| platelet alpha granule membrane | GO:0031092 | Cell Component | 4 | 0.013113 |
| plasma membrane | GO:0005886 | Cell Component | 91 | 0.013578 |
| endocytic vesicle lumen | GO:0071682 | Cell Component | 4 | 0.025993 |
| specific granule membrane | GO:0035579 | Cell Component | 7 | 0.028065 |
| extracellular vesicle | GO:1903561 | Cell Component | 44 | 0.029886 |
| azurophil granule lumen | GO:0035578 | Cell Component | 7 | 0.030115 |
| extracellular organelle | GO:0043230 | Cell Component | 44 | 0.031538 |
| extracellular exosome | GO:0070062 | Cell Component | 43 | 0.048576 |
| anchored component of membrane | GO:0031225 | Cell Component | 9 | 0.049565 |

**Supplemental Table S2C. Differentially expressed genes (N=202) in the VDAART subjects with all conditions (N=15) versus subjects with none of the conditions (N=13) that appeared in the overlapping gene set of initial analysis (N=238).**

|  | **Gene Symbol** | **Probe ID** | **P-Value** | **Fold Change** | **Regulation** | **LCC** |
| --- | --- | --- | --- | --- | --- | --- |
| 1 | AHSP | 7995237 | 0.0001852 | 0.9605 | Down | Yes |
| 2 | AK5 | 7902452 | 0.0007524 | 1.024 | Up | Yes |
| 3 | ALOX15 | 8011680 | 0.01916 | 0.9761 | Down | Yes |
| 4 | ANKRD22 | 7934898 | 0.0007843 | 0.9994 | Down | Yes |
| 5 | ARG1 | 8122058 | 0.01114 | 0.9784 | Down | Yes |
| 6 | ARRDC4 | 7986350 | 0.03588 | 1.004 | Up | No |
| 7 | BANK1 | 8096617 | 0.01906 | 0.9883 | Down | Yes |
| 8 | BPGM | 8136341 | 0.0001704 | 0.9639 | Down | Yes |
| 9 | BPI | 8062444 | 0.0005308 | 0.9905 | Down | Yes |
| 10 | C17orf97 | 8003607 | 0.005289 | 0.9771 | Down | Yes |
| 11 | C3AR1 | 7960874 | 0.0004115 | 1.016 | Up | Yes |
| 12 | C4BPA | 7909318 | 2.36E-10 | 0.9046 | Down | Yes |
| 13 | CA2 | 8147132 | 0.03725 | 1.01 | Up | Yes |
| 14 | CASP5 | 7951385 | 0.004476 | 0.9797 | Down | Yes |
| 15 | CC2D2B | 7929533 | 0.04964 | 0.9917 | Down | No |
| 16 | CCR3 | 8079383 | 0.01112 | 0.9888 | Down | Yes |
| 17 | CD160 | 7919243 | 0.0002878 | 0.9967 | Down | Yes |
| 18 | CD177 | 8029280 | 1.55E-11 | 0.9202 | Down | Yes |
| 19 | CD24 | 8177222 | 6.16E-06 | 0.9603 | Down | Yes |
| 20 | CD274 | 8154233 | 0.0009454 | 0.9734 | Down | Yes |
| 21 | CD93 | 8065359 | 0.003265 | 1.029 | Up | Yes |
| 22 | CEACAM6 | 8029098 | 0.0008228 | 0.9931 | Down | Yes |
| 23 | CENPK | 8112376 | 1.11E-07 | 0.9515 | Down | No |
| 24 | CEP78 | 8156026 | 0.002599 | 0.9741 | Down | Yes |
| 25 | CFD | 8024062 | 0.0003576 | 1.01 | Up | Yes |
| 26 | CHI3L1 | 7923547 | 3.20E-11 | 1.075 | Up | Yes |
| 27 | CKS2 | 8156290 | 1.27E-06 | 0.9432 | Down | No |
| 28 | CLC | 8036755 | 0.0006468 | 0.9738 | Down | Yes |
| 29 | CLEC12A | 7953901 | 1.88E-07 | 1.038 | Up | Yes |
| 30 | CLEC12B | 7953914 | 5.40E-08 | 1.044 | Up | Yes |
| 31 | CLEC4C | 7960832 | 3.14E-05 | 1.037 | Up | Yes |
| 32 | CLEC4D | 7953749 | 0.007277 | 0.9784 | Down | Yes |
| 33 | CLU | 8149927 | 0.02246 | 0.985 | Down | Yes |
| 34 | CMBL | 8110971 | 0.03859 | 0.9987 | Down | No |
| 35 | CPA3 | 8083260 | 4.08E-08 | 1.056 | Up | Yes |
| 36 | CPT1A | 7949971 | 0.003207 | 0.9747 | Down | No |
| 37 | CRISP3 | 8126905 | 6.80E-09 | 0.9512 | Down | Yes |
| 38 | CSGALNACT1 | 8149574 | 0.002085 | 1.018 | Up | No |
| 39 | CTSW | 7941444 | 0.0494 | 0.9838 | Down | Yes |
| 40 | CXCL10 | 8101126 | 1.07E-06 | 0.9247 | Down | Yes |
| 41 | CYP1B1 | 8051583 | 0.01506 | 1.013 | Up | Yes |
| 42 | CYP27A1 | 8048432 | 0.002226 | 1.014 | Up | No |
| 43 | DEFA4 | 8149109 | 0.0017 | 0.9771 | Down | Yes |
| 44 | DHRS9 | 8046124 | 0.000153 | 1.016 | Up | No |
| 45 | DOCK4 | 8142345 | 0.02444 | 0.9862 | Down | No |
| 46 | DSC2 | 8022711 | 2.70E-10 | 1.075 | Up | Yes |
| 47 | E2F2 | 7913644 | 0.01749 | 0.9883 | Down | Yes |
| 48 | ECRP | 7973108 | 0.04699 | 1.013 | Up | Not Mapped |
| 49 | ELOVL7 | 8112274 | 0.04771 | 0.989 | Down | No |
| 50 | ENC1 | 8112615 | 0.003237 | 1.002 | Up | Yes |
| 51 | ENKUR | 7932598 | 0.0008632 | 0.9645 | Down | No |
| 52 | ERICH1 | 8148978 | 0.000124 | 1.034 | Up | No |
| 53 | F2RL1 | 8106403 | 0.01812 | 1.017 | Up | Yes |
| 54 | FAM3B | 8068684 | 0.04151 | 1.005 | Up | No |
| 55 | FCER1A | 7906443 | 0.00135 | 1.021 | Up | Yes |
| 56 | FCRL5 | 7921237 | 0.009457 | 1.007 | Up | Yes |
| 57 | FGFBP2 | 8099471 | 2.16E-05 | 0.9648 | Down | Yes |
| 58 | FLVCR1 | 7909628 | 0.04701 | 1.016 | Up | Yes |
| 59 | FMN1 | 7987145 | 9.82E-09 | 0.929 | Down | Yes |
| 60 | FOLR3 | 7942328 | 0.0003371 | 0.9738 | Down | Yes |
| 61 | GM2A | 8109344 | 0.01303 | 0.9824 | Down | Yes |
| 62 | GNG11 | 8134257 | 1.10E-08 | 0.9249 | Down | Yes |
| 63 | GNLY | 8043236 | 0.000534 | 0.9673 | Down | Yes |
| 64 | GPR146 | 8131067 | 1.73E-05 | 0.9525 | Down | No |
| 65 | GRAMD1C | 8081758 | 0.02207 | 0.9873 | Down | No |
| 66 | GSTM1 | 7903765 | 3.54E-07 | 0.9646 | Down | Yes |
| 67 | GSTT1 | 8074980 | 0.008201 | 1.013 | Up | Not Mapped |
| 68 | GYPA | 8102998 | 0.0001952 | 0.9633 | Down | Yes |
| 69 | GYPB | 8102993 | 1.94E-05 | 1.042 | Up | Yes |
| 70 | GYPE | 8102988 | 0.002493 | 0.9726 | Down | Yes |
| 71 | GZMH | 7978360 | 0.0002458 | 0.9659 | Down | Yes |
| 72 | GZMK | 8105331 | 0.003646 | 0.9775 | Down | Yes |
| 73 | HBM | 7991758 | 0.01867 | 0.9859 | Down | Yes |
| 74 | HBZ | 7991750 | 0.01053 | 0.931 | Down | Yes |
| 75 | HEMGN | 8162719 | 0.01222 | 0.9815 | Down | Yes |
| 76 | HIP1 | 8140319 | 0.0007533 | 1.025 | Up | No |
| 77 | HLA-DOB | 8178833 | 0.04273 | 0.9881 | Down | Yes |
| 78 | HLA-DPA1 | 8178891 | 6.03E-05 | 1.041 | Up | Yes |
| 79 | HLA-DPB1 | 8179519 | 0.008236 | 1.023 | Up | Yes |
| 80 | HLA-DQB1 | 8125461 | 0.002106 | 1.027 | Up | Yes |
| 81 | HORMAD1 | 7919787 | 0.006995 | 0.9835 | Down | No |
| 82 | HP | 7997188 | 7.32E-05 | 1.001 | Up | Yes |
| 83 | HRH4 | 8020684 | 0.0006535 | 0.9868 | Down | Yes |
| 84 | IDO1 | 8146092 | 0.002463 | 0.9714 | Down | Yes |
| 85 | IFI27 | 7976443 | 0.02953 | 0.9897 | Down | Yes |
| 86 | IFI44 | 7902553 | 7.80E-07 | 1.006 | Up | Yes |
| 87 | IFI44L | 7902541 | 1.81E-06 | 1.004 | Up | Yes |
| 88 | IFI6 | 7914127 | 0.005224 | 1.004 | Up | Yes |
| 89 | IFIT1 | 7929065 | 0.001557 | 1.017 | Up | Yes |
| 90 | IGF2BP2 | 8092552 | 0.003832 | 0.9839 | Down | No |
| 91 | IL18RAP | 8044049 | 0.0471 | 1 | Down | Yes |
| 92 | IL1R1 | 8043995 | 0.003793 | 0.9786 | Down | Yes |
| 93 | IL23R | 7902189 | 0.0002933 | 1.019 | Up | Yes |
| 94 | IL5RA | 8085062 | 0.00799 | 0.975 | Down | Yes |
| 95 | ITGA2B | 8016044 | 0.01378 | 0.9834 | Down | Yes |
| 96 | ITGB3 | 8007931 | 0.006602 | 0.9858 | Down | Yes |
| 97 | JUP | 8015412 | 4.30E-05 | 1.041 | Up | Yes |
| 98 | KEL | 8143534 | 0.003113 | 1.012 | Up | Yes |
| 99 | KIAA0825 | 8113097 | 0.0007227 | 0.9677 | Down | No |
| 100 | KIAA1324 | 7903592 | 0.001976 | 0.984 | Down | No |
| 101 | KIAA1324L | 8140709 | 0.001848 | 1.02 | Up | No |
| 102 | KLRF1 | 7953892 | 1.32E-06 | 0.9707 | Down | Yes |
| 103 | KLRG1 | 7953835 | 0.02043 | 1.009 | Up | Yes |
| 104 | KRT23 | 8015133 | 0.04886 | 1.01 | Up | Yes |
| 105 | KRT73 | 7963471 | 0.004986 | 0.9774 | Down | Yes |
| 106 | LCN2 | 8158167 | 5.98E-06 | 0.9629 | Down | Yes |
| 107 | LGALS2 | 8075956 | 0.0156 | 1.005 | Up | No |
| 108 | LILRA3 | 8039226 | 2.99E-05 | 1.04 | Up | Not Mapped |
| 109 | LINC00189 | 8068100 | 0.01651 | 1.039 | Up | Not Mapped |
| 110 | LINC00328 | 7904478 | 0.0001296 | 1.02 | Up | Not Mapped |
| 111 | LRRC6 | 8152962 | 4.19E-10 | 1.065 | Up | No |
| 112 | LRRN1 | 8077366 | 8.93E-09 | 1.063 | Up | No |
| 113 | LRRN3 | 8135488 | 0.01853 | 1.007 | Up | No |
| 114 | LTF | 8086607 | 5.28E-05 | 0.9771 | Down | Yes |
| 115 | MFSD9 | 8054356 | 0.003584 | 0.9858 | Down | No |
| 116 | MILR1 | 8009243 | 0.01358 | 0.9861 | Down | No |
| 117 | MIR646HG | 8063793 | 3.14E-07 | 1.042 | Up | Not Mapped |
| 118 | MMP8 | 7951246 | 1.65E-11 | 0.9301 | Down | Yes |
| 119 | MMRN1 | 8096415 | 2.50E-05 | 0.958 | Down | Yes |
| 120 | MPZL2 | 7952046 | 0.000152 | 1.033 | Up | Yes |
| 121 | MS4A3 | 7940216 | 0.0004419 | 0.9681 | Down | Yes |
| 122 | MX1 | 8068713 | 1.41E-06 | 1.027 | Up | Yes |
| 123 | MYBL1 | 8151101 | 0.01217 | 0.9842 | Down | No |
| 124 | MYL4 | 8007921 | 0.0002416 | 0.9636 | Down | Yes |
| 125 | NDUFB3 | 8047372 | 0.0004492 | 0.9677 | Down | No |
| 126 | NEBL | 7932453 | 0.007917 | 0.9921 | Down | No |
| 127 | NFXL1 | 8100179 | 0.000948 | 1.002 | Up | No |
| 128 | NIPAL2 | 8151952 | 8.78E-05 | 1.039 | Up | No |
| 129 | NMRK1 | 8161839 | 2.59E-05 | 0.9554 | Down | No |
| 130 | NOD2 | 7995539 | 0.002892 | 0.9848 | Down | Yes |
| 131 | NPCDR1 | 8088468 | 0.001584 | 1.016 | Up | Not Mapped |
| 132 | NRG1 | 8145766 | 0.0001739 | 0.9728 | Down | Yes |
| 133 | NT5E | 8120967 | 0.0008768 | 0.9763 | Down | Yes |
| 134 | OLFM4 | 7969288 | 0.001967 | 0.9588 | Down | Yes |
| 135 | OMG | 8014057 | 8.28E-06 | 0.9389 | Down | No |
| 136 | OR52K3P | 7937940 | 0.01481 | 1.011 | Up | Not Mapped |
| 137 | ORAI2 | 8135172 | 0.00203 | 1.029 | Up | No |
| 138 | ORM1 | 8157446 | 4.75E-16 | 1.133 | Up | Yes |
| 139 | OSBPL10 | 8085984 | 0.03928 | 1.014 | Up | No |
| 140 | P2RX1 | 8011499 | 0.01854 | 1.011 | Up | Yes |
| 141 | P2RY12 | 8091530 | 0.02949 | 0.9904 | Down | Yes |
| 142 | PADI2 | 7912937 | 6.78E-06 | 1.043 | Up | Yes |
| 143 | PAM | 8107133 | 1.70E-05 | 1.037 | Up | No |
| 144 | PAPSS1 | 8102214 | 2.52E-06 | 1.039 | Up | Yes |
| 145 | PAX8-AS1 | 8044605 | 0.0002004 | 0.9729 | Down | Not Mapped |
| 146 | PDK4 | 8141094 | 0.001113 | 0.9801 | Down | No |
| 147 | PDZK1IP1 | 7915910 | 0.03246 | 1.013 | Up | Yes |
| 148 | PITHD1 | 7898894 | 0.005456 | 0.9862 | Down | No |
| 149 | PLA2G7 | 8126784 | 2.09E-05 | 1.03 | Up | Yes |
| 150 | PLB1 | 8041061 | 0.01965 | 0.997 | Down | Yes |
| 151 | PLEK2 | 7979710 | 0.04181 | 1.006 | Up | No |
| 152 | PPBP | 8100971 | 0.00819 | 0.9764 | Down | Yes |
| 153 | PRKAR2B | 8135378 | 0.005447 | 0.9802 | Down | Yes |
| 154 | PVALB | 8075838 | 5.58E-06 | 1.043 | Up | Yes |
| 155 | RAP1GAP | 7913385 | 0.01364 | 1.012 | Up | Yes |
| 156 | RHD | 7898998 | 2.52E-05 | 0.9632 | Down | Yes |
| 157 | RNASE2 | 7973110 | 0.001949 | 0.9766 | Down | Yes |
| 158 | RNASE3 | 7973105 | 5.34E-06 | 0.9553 | Down | Yes |
| 159 | RNF182 | 8116980 | 4.46E-12 | 0.9043 | Down | No |
| 160 | RPL7 | 8109222 | 1.69E-07 | 0.9341 | Down | No |
| 161 | RPS23 | 8112961 | 0.04053 | 0.984 | Down | No |
| 162 | RSAD2 | 8040080 | 0.0005634 | 1.011 | Up | Yes |
| 163 | RUNDC3A | 8007607 | 0.04289 | 1.011 | Up | No |
| 164 | S100B | 8071036 | 0.0006456 | 0.9962 | Down | Yes |
| 165 | S100P | 8093950 | 0.02522 | 1.012 | Up | Yes |
| 166 | SAMD12 | 8152506 | 0.01031 | 0.9886 | Down | No |
| 167 | SELP | 7922200 | 0.03383 | 0.9897 | Down | Yes |
| 168 | SERPINB10 | 8021645 | 2.59E-09 | 0.9252 | Down | No |
| 169 | SERPING1 | 7940028 | 6.35E-09 | 1.052 | Up | Yes |
| 170 | SH2D1B | 7921900 | 0.01121 | 0.99 | Down | Yes |
| 171 | SH3BGRL2 | 8120833 | 0.005231 | 0.9792 | Down | No |
| 172 | SLC12A7 | 8110755 | 0.007741 | 1.018 | Up | No |
| 173 | SLC14A1 | 8021081 | 0.0006677 | 0.9728 | Down | Yes |
| 174 | SLC38A11 | 8056363 | 0.001555 | 1.012 | Up | No |
| 175 | SLPI | 8066493 | 1.95E-05 | 0.9519 | Down | Yes |
| 176 | SMOX | 8060745 | 0.04833 | 0.9985 | Down | Yes |
| 177 | SNORA68 | 8026875 | 0.01786 | 0.9915 | Down | Not Mapped |
| 178 | SPARC | 8115327 | 0.008434 | 0.9786 | Down | Yes |
| 179 | TARP | 8139125 | 0.04816 | 0.9936 | Down | Not Mapped |
| 180 | TCL1A | 7981183 | 0.0004812 | 0.966 | Down | Yes |
| 181 | TCN1 | 7948444 | 4.77E-05 | 0.974 | Down | Yes |
| 182 | THBS1 | 7982597 | 0.02134 | 0.9919 | Down | Yes |
| 183 | TMEM144 | 8098041 | 0.001452 | 1.014 | Up | No |
| 184 | TMEM176A | 8137264 | 2.70E-10 | 1.051 | Up | Yes |
| 185 | TMEM176B | 8143790 | 2.79E-06 | 1.032 | Up | Yes |
| 186 | TMTC1 | 7962058 | 0.0003741 | 0.97 | Down | No |
| 187 | TNFAIP6 | 8045688 | 0.008142 | 1.004 | Up | Yes |
| 188 | TPTEP1 | 8071063 | 4.33E-05 | 0.9611 | Down | Not Mapped |
| 189 | TREML1 | 8126269 | 0.0004467 | 0.9664 | Down | Yes |
| 190 | TREML3P | 8126296 | 0.02358 | 0.9948 | Down | Not Mapped |
| 191 | TREML4 | 8119427 | 0.01249 | 0.9876 | Down | Yes |
| 192 | TUBB2A | 8123644 | 9.52E-13 | 0.9031 | Down | Yes |
| 193 | UBE2O | 8018708 | 0.008367 | 0.9882 | Down | No |
| 194 | USP53 | 8097098 | 0.007823 | 0.9935 | Down | No |
| 195 | UTS2 | 7912136 | 7.02E-06 | 0.9148 | Down | Yes |
| 196 | VNN1 | 8129618 | 0.04699 | 0.9647 | Down | Yes |
| 197 | VNN3 | 8129627 | 0.01472 | 1.013 | Up | Yes |
| 198 | VSTM1 | 8039109 | 0.002256 | 0.978 | Down | No |
| 199 | WLS | 7916862 | 0.00207 | 1.017 | Up | Yes |
| 200 | XCL1 | 7907156 | 1.42E-05 | 0.9536 | Down | Yes |
| 201 | YOD1 | 7909283 | 0.0008269 | 0.9645 | Down | No |
| 202 | ZNF763 | 8025978 | 0.004339 | 1.014 | Up | Not Mapped |

**Supplemental Table S2D. Overlapping gene signatures enriched in placentas from uncomplicated pregnancies (1st trimester vs 2nd trimester)**

|  | **Gene Symbol** | **Probe ID** | **P-value** | **Fold Change** | **Regulation** |
| --- | --- | --- | --- | --- | --- |
| 1 | AHSP | 219672_at | 0.02253 | 0.98 | Down |
| 2 | ANKRD22 | 239196_at | 0.005227 | 1.05 | Up |
| 3 | ARG1 | 206177_s_at | 0.001125 | 1.02 | Up |
| 4 | BANK1 | 222915_s_at | 0.0219 | 1.06 | Up |
| 5 | C3AR1 | 209906_at | 0.00018 | 1.01 | Up |
| 6 | C4BPA | 205654_at | 5.64E-08 | 1.08 | Up |
| 7 | CA2 | 209301_at | 0.007034 | 0.93 | Down |
| 8 | CD24 | 216379_x_at | 0.001074 | 1.04 | Up |
| 9 | CD274 | 227458_at | 0.007994 | 1.02 | Up |
| 10 | CD93 | 202878_s_at | 1.24E-06 | 0.98 | Down |
| 11 | CEP78 | 1557985_s_at | 0.02155 | 0.96 | Down |
| 12 | CFD | 205382_s_at | 0.007964 | 0.99 | Down |
| 13 | CHI3L1 | 209396_s_at | 0.009673 | 1.14 | Up |
| 14 | CKS2 | 204170_s_at | 0.006611 | 1.05 | Up |
| 15 | CLEC4D | 1552773_at | 0.004709 | 1.01 | Up |
| 16 | CLU | 208791_at | 3.92E-09 | 0.94 | Down |
| 17 | CMBL | 227522_at | 0.000643 | 0.99 | Down |
| 18 | CPT1A | 203634_s_at | 0.009186 | 1.01 | Up |
| 19 | CSGALNACT1 | 219049_at | 0.002909 | 1.06 | Up |
| 20 | CTSW | 214450_at | 0.02567 | 0.94 | Down |
| 21 | CXCL10 | 204533_at | 0.03557 | 0.94 | Down |
| 22 | CYP1B1 | 202434_s_at | 0.02205 | 1.01 | Up |
| 23 | CYP27A1 | 203979_at | 0.000633 | 0.97 | Down |
| 24 | DHRS9 | 223952_x_at | 0.02943 | 1.02 | Up |
| 25 | DOCK4 | 205003_at | 3.73E-05 | 0.95 | Down |
| 26 | DSC2 | 204750_s_at | 4.34E-06 | 1.13 | Up |
| 27 | E2F2 | 228361_at | 0.001386 | 1.03 | Up |
| 28 | ELOVL7 | 227180_at | 0.000649 | 0.94 | Down |
| 29 | F2RL1 | 213506_at | 1.80E-08 | 1.06 | Up |
| 30 | FAM160A1 | 242687_at | 0.01458 | 1.01 | Up |
| 31 | FCER1A | 211734_s_at | 0.02703 | 1.04 | Up |
| 32 | FCGR2B | 210889_s_at | 3.66E-12 | 1.02 | Up |
| 33 | GATM | 203178_at | 0.000209 | 1.06 | Up |
| 34 | GBP5 | 229625_at | 0.02918 | 1.01 | Up |
| 35 | GM2A | 215891_s_at | 0.007791 | 0.98 | Down |
| 36 | GNG11 | 239942_at | 3.61E-10 | 0.89 | Down |
| 37 | GNLY | 37145_at | 0.02294 | 0.96 | Down |
| 38 | GPR146 | 228770_at | 0.01889 | 0.96 | Down |
| 39 | GRAMD1C | 219313_at | 7.43E-05 | 1.05 | Up |
| 40 | GSTM1 | 215333_x_at | 0.005492 | 1.28 | Up |
| 41 | GSTT1 | 203815_at | 0.004666 | 0.96 | Down |
| 42 | GTSF1 | 227711_at | 0.005997 | 1.03 | Up |
| 43 | GYPA | 211820_x_at | 0.003354 | 1.02 | Up |
| 44 | GYPB | 216833_x_at | 0.004796 | 1.07 | Up |
| 45 | GZMH | 210321_at | 0.001214 | 0.97 | Down |
| 46 | GZMK | 206666_at | 0.000128 | 0.98 | Down |
| 47 | HBZ | 206647_at | 6.37E-08 | 0.89 | Down |
| 48 | HIP1 | 205425_at | 3.09E-05 | 0.99 | Down |
| 49 | HLA-DPA1 | 211991_s_at | 1.29E-06 | 1.12 | Up |
| 50 | HLA-DPB1 | 201137_s_at | 0.000438 | 1.07 | Up |
| 51 | HLA-DQB1 | 211654_x_at | 3.03E-05 | 1.05 | Up |
| 52 | HP | 206697_s_at | 0.02834 | 1.09 | Up |
| 53 | IDO1 | 210029_at | 2.46E-11 | 1.05 | Up |
| 54 | IFI27 | 202411_at | 0.04334 | 1.04 | Up |
| 55 | IFI44 | 214453_s_at | 0.000112 | 1.01 | Up |
| 56 | IFI44L | 204439_at | 0.003676 | 1.02 | Up |
| 57 | IFIT1 | 203153_at | 0.005907 | 1.09 | Up |
| 58 | IGF2BP2 | 223963_s_at | 0.006938 | 0.95 | Down |
| 59 | IGKC | 214768_x_at | 0.03956 | 1.10 | Up |
| 60 | IL1R1 | 215561_s_at | 0.01672 | 0.96 | Down |
| 61 | IL5RA | 207902_at | 0.03294 | 1.01 | Up |
| 62 | JUP | 201015_s_at | 7.51E-06 | 1.03 | Up |
| 63 | KIAA1324 | 221874_at | 0.001931 | 0.81 | Down |
| 64 | KIAA1324L | 244317_at | 0.01217 | 0.96 | Down |
| 65 | KRT23 | 218963_s_at | 0.003815 | 0.99 | Down |
| 66 | LCN2 | 212531_at | 9.40E-05 | 0.97 | Down |
| 67 | LGALSL | 226188_at | 1.09E-05 | 0.98 | Down |
| 68 | LPAR1 | 204036_at | 0.000102 | 0.91 | Down |
| 69 | LRRC6 | 206483_at | 0.03915 | 1.03 | Up |
| 70 | MASTL | 228468_at | 0.005895 | 0.97 | Down |
| 71 | MFSD9 | 213393_at | 0.0178 | 1.01 | Up |
| 72 | MILR1 | 217513_at | 0.02152 | 1.01 | Up |
| 73 | MMRN1 | 205612_at | 0.004182 | 0.89 | Down |
| 74 | MYBL1 | 213906_at | 0.006722 | 0.95 | Down |
| 75 | NEBL | 203961_at | 0.004262 | 1.10 | Up |
| 76 | NIPAL2 | 227001_at | 0.000169 | 0.95 | Down |
| 77 | NLRP3 | 207075_at | 0.001317 | 0.95 | Down |
| 78 | NMRK1 | 219147_s_at | 0.02376 | 0.96 | Down |
| 79 | NOD2 | 220066_at | 0.004071 | 0.87 | Down |
| 80 | NRG1 | 208232_x_at | 0.04146 | 0.93 | Down |
| 81 | NT5E | 1553995_a_at | 0.02202 | 0.99 | Down |
| 82 | OLFM4 | 212768_s_at | 1.05E-05 | 1.05 | Up |
| 83 | ORAI2 | 1558426_x_at | 0.001609 | 1.02 | Up |
| 84 | OSBPL10 | 219073_s_at | 0.005957 | 1.05 | Up |
| 85 | P2RY12 | 235885_at | 0.009342 | 0.94 | Down |
| 86 | PAM | 202336_s_at | 0.003774 | 1.05 | Up |
| 87 | PAPSS1 | 209043_at | 0.009693 | 1.04 | Up |
| 88 | PDK4 | 205960_at | 5.87E-06 | 1.04 | Up |
| 89 | PDZK1IP1 | 1553589_a_at | 0.000199 | 1.08 | Up |
| 90 | PLA2G7 | 206214_at | 0.000465 | 0.94 | Down |
| 91 | PPBP | 214146_s_at | 1.71E-06 | 0.90 | Down |
| 92 | PRKAR2B | 203680_at | 1.46E-06 | 0.88 | Down |
| 93 | PTGS1 | 238669_at | 0.02745 | 0.96 | Down |
| 94 | PTPRM | 1555579_s_at | 0.03807 | 0.97 | Down |
| 95 | PVALB | 205336_at | 0.005329 | 1.05 | Up |
| 96 | RAP1GAP | 203911_at | 4.38E-05 | 0.94 | Down |
| 97 | RHD | 210430_x_at | 0.006033 | 0.95 | Down |
| 98 | RPS23 | 227722_at | 0.02664 | 0.93 | Down |
| 99 | RSAD2 | 213797_at | 0.000246 | 1.04 | Up |
| 100 | SAMD12 | 238673_at | 0.01239 | 1.01 | Up |
| 101 | SELP | 206049_at | 0.02483 | 0.95 | Down |
| 102 | SERPING1 | 200986_at | 0.03487 | 1.06 | Up |
| 103 | SH2D1B | 1553177_at | 0.003122 | 0.95 | Down |
| 104 | SLPI | 203021_at | 9.61E-09 | 0.98 | Down |
| 105 | SMOX | 1555680_a_at | 0.01056 | 0.99 | Down |
| 106 | THBD | 237252_at | 0.01017 | 0.99 | Down |
| 107 | THBS1 | 201109_s_at | 0.01319 | 0.95 | Down |
| 108 | TMEM144 | 228624_at | 5.37E-05 | 0.96 | Down |
| 109 | TMEM176A | 218345_at | 7.30E-05 | 1.03 | Up |
| 110 | TMEM176B | 220532_s_at | 3.20E-08 | 1.01 | Up |
| 111 | TMTC1 | 226931_at | 0.004503 | 0.91 | Down |
| 112 | TNFAIP6 | 206026_s_at | 0.000303 | 0.98 | Down |
| 113 | TUBB2A | 204141_at | 0.009335 | 0.82 | Down |
| 114 | USP53 | 237465_at | 0.001531 | 1.02 | Up |
| 115 | VNN1 | 205844_at | 0.01321 | 1.12 | Up |
| 116 | VNN3 | 220528_at | 0.04448 | 1.01 | Up |
| 117 | WLS | 228949_at | 0.005289 | 0.95 | Down |
| 118 | XCL1 | 206366_x_at | 0.01822 | 1.02 | Up |
| 119 | YOD1 | 215150_at | 0.02036 | 0.96 | Down |
| 120 | ZDHHC2 | 222731_at | 0.04472 | 1.06 | Up |
| 121 | ZNF641 | 229897_at | 0.002255 | 0.97 | Down |

**Supplemental Table S2E. Overlapping gene signatures enriched in placenta (pregnancies with preeclampsia vs uncomplicated pregnancies)**

|  | **Gene Symbol** | **Probe ID** | **P-value** | **Fold Change** | **Regulation** |
| --- | --- | --- | --- | --- | --- |
| 1 | ARRDC4 | 7986350 | 0.002138 | 1.09 | Up |
| 2 | BPI | 8062444 | 0.03281 | 1.07 | Up |
| 3 | C17orf97 | 8003607 | 0.0443 | 1.02 | Up |
| 4 | C3AR1 | 7960874 | 2.56E-11 | 1.20 | Up |
| 5 | CA2 | 8147132 | 1.69E-10 | 0.88 | Down |
| 6 | CAT | 7939298 | 0.005511 | 1.08 | Up |
| 7 | CC2D2B | 7929541 | 0.005278 | 0.98 | Down |
| 8 | CD177 | 8037298 | 6.98E-13 | 1.19 | Up |
| 9 | CHI3L1 | 7923547 | 0.04068 | 0.98 | Down |
| 10 | CKS2 | 8156290 | 4.65E-07 | 0.92 | Down |
| 11 | CLC | 8036755 | 1.08E-25 | 0.77 | Down |
| 12 | CLEC4D | 7953749 | 1.54E-05 | 1.12 | Up |
| 13 | CLU | 8149927 | 0.000191 | 0.92 | Down |
| 14 | CPA3 | 8083260 | 0.001187 | 1.10 | Up |
| 15 | CPT1A | 7949971 | 0.02014 | 1.05 | Up |
| 16 | CRISP3 | 8126905 | 0.003605 | 1.08 | Up |
| 17 | CYBRD1 | 8046333 | 0.000172 | 1.10 | Up |
| 18 | DEFA4 | 8149109 | 0.02966 | 1.07 | Up |
| 19 | ENKUR | 7932598 | 0.002243 | 0.92 | Down |
| 20 | ERICH1 | 8148978 | 3.98E-07 | 1.13 | Up |
| 21 | F2RL1 | 8106403 | 0.000673 | 0.91 | Down |
| 22 | FAM160A1 | 8097801 | 9.27E-12 | 0.85 | Down |
| 23 | FAM3B | 8068684 | 1.53E-07 | 0.9 | Down |
| 24 | FMN1 | 7987145 | 3.48E-09 | 0.85 | Down |
| 25 | GATM | 7988414 | 0.000177 | 1.11 | Up |
| 26 | GNG11 | 8134257 | 3.68E-05 | 1.12 | Up |
| 27 | GNLY | 8043236 | 0.0492 | 0.96 | Down |
| 28 | GPR146 | 8131067 | 2.97E-37 | 0.67 | Down |
| 29 | GSTT1 | 8074980 | 0.008967 | 0.97 | Down |
| 30 | HBZ | 7991750 | 0.00018 | 0.94 | Down |
| 31 | HLA-DOB | 8125470 | 0.04457 | 0.99 | Down |
| 32 | HLA-DPB1 | 8118594 | 0.000196 | 0.93 | Down |
| 33 | HLA-DQB1 | 8125461 | 0.008977 | 0.97 | Down |
| 34 | HP | 7997188 | 0.02355 | 1.05 | Up |
| 35 | IDO1 | 8146092 | 2.84E-19 | 1.30 | Up |
| 36 | IFI27 | 7976443 | 1.22E-05 | 0.89 | Down |
| 37 | IFI44 | 7902553 | 0.000349 | 0.94 | Down |
| 38 | IFI44L | 7902541 | 2.49E-16 | 0.81 | Down |
| 39 | IFIT1 | 7929065 | 9.31E-14 | 0.83 | Down |
| 40 | IGKC | 8161563 | 0.0105 | 1.05 | Up |
| 41 | ITGB3 | 8007931 | 0.0408 | 1.02 | Up |
| 42 | JUP | 8015412 | 1.08E-05 | 0.88 | Down |
| 43 | KEL | 8143534 | 1.80E-17 | 0.81 | Down |
| 44 | KIAA0825 | 8113103 | 2.89E-06 | 1.14 | Up |
| 45 | KIAA1324L | 8140709 | 0.000251 | 1.10 | Up |
| 46 | LINC00189 | 8068100 | 9.16E-06 | 0.91 | Down |
| 47 | LINC00328 | 8071049 | 8.53E-08 | 1.10 | Up |
| 48 | LPAR1 | 8163257 | 0.000486 | 1.11 | Up |
| 49 | LRRC6 | 8152962 | 0.01155 | 1.08 | Up |
| 50 | LRRN1 | 8077366 | 1.08E-05 | 1.01 | Up |
| 51 | LRRN3 | 8135488 | 5.40E-27 | 1.39 | Up |
| 52 | LTF | 8086607 | 2.83E-27 | 0.74 | Down |
| 53 | MILR1 | 8009243 | 1.32E-09 | 1.19 | Up |
| 54 | MMP8 | 7951246 | 5.30E-08 | 1.14 | Up |
| 55 | MS4A3 | 7940216 | 8.60E-08 | 1.15 | Up |
| 56 | MX1 | 8068713 | 6.02E-10 | 0.86 | Down |
| 57 | MYBL1 | 8151101 | 0.02638 | 1.06 | Up |
| 58 | NEBL | 7932453 | 4.53E-05 | 0.89 | Down |
| 59 | NQO2 | 8116610 | 0.000103 | 1.11 | Up |
| 60 | NT5E | 8120967 | 1.24E-06 | 0.9 | Down |
| 61 | ODC1 | 8050240 | 0.007769 | 1.09 | Up |
| 62 | OLFM4 | 7969288 | 8.25E-05 | 1.09 | Up |
| 63 | OR52K3P | 7937940 | 4.84E-05 | 0.90 | Down |
| 64 | P2RY12 | 8091530 | 3.52E-08 | 1.14 | Up |
| 65 | PAM | 8107133 | 8.58E-10 | 0.85 | Down |
| 66 | PAX8-AS1 | 8044605 | 3.32E-05 | 0.92 | Down |
| 67 | PDK4 | 8141094 | 0.002394 | 1.08 | Up |
| 68 | PDZK1IP1 | 7915910 | 0.01535 | 0.93 | Down |
| 69 | PPBP | 8100971 | 0.000371 | 1.05 | Up |
| 70 | PRRG4 | 7939150 | 0.002015 | 0.93 | Down |
| 71 | RAP1GAP | 7913385 | 0.000872 | 1.10 | Up |
| 72 | RNASE2 | 7973110 | 0.03063 | 0.97 | Down |
| 73 | RPS23 | 8112961 | 0.006102 | 1.07 | Up |
| 74 | RSAD2 | 8040080 | 0.00821 | 0.94 | Down |
| 75 | SELP | 7922200 | 2.74E-05 | 1.11 | Up |
| 76 | SERPINB10 | 8021645 | 7.31E-12 | 1.21 | Up |
| 77 | SERPINB9P1 | 8123606 | 1.25E-05 | 0.91 | Down |
| 78 | SERPING1 | 7940028 | 8.76E-08 | 0.87 | Down |
| 79 | SH3BGRL2 | 8120833 | 7.46E-05 | 1.13 | Up |
| 80 | SLPI | 8066493 | 2.08E-05 | 0.92 | Down |
| 81 | ST6GALNAC4 | 8164314 | 0.03192 | 0.95 | Down |
| 82 | TARP | 8139128 | 0.009767 | 1.04 | Up |
| 83 | THBD | 8065353 | 0.01859 | 0.96 | Down |
| 84 | THBS1 | 7982597 | 0.000375 | 1.05 | Up |
| 85 | TMEM144 | 8098041 | 1.13E-05 | 1.14 | Up |
| 86 | TMEM176A | 8137264 | 6.09E-12 | 1.20 | Up |
| 87 | TMEM176B | 8143790 | 1.34E-05 | 1.14 | Up |
| 88 | TMTC1 | 7962058 | 2.93E-08 | 0.90 | Down |
| 89 | TNFAIP6 | 8045688 | 0.04143 | 0.96 | Down |
| 90 | TPTEP1 | 8071063 | 2.70E-05 | 0.92 | Down |
| 91 | USP53 | 8097098 | 0.02943 | 0.93 | Down |
| 92 | VNN1 | 8129618 | 1.40E-13 | 1.20 | Up |
| 93 | VNN3 | 8129627 | 3.90E-44 | 1.60 | Up |
| 94 | WLS | 7916862 | 2.83E-05 | 1.12 | Up |
| 95 | XCL1 | 7907156 | 1.11E-17 | 1.22 | Up |
| 96 | YOD1 | 7923967 | 0.02735 | 0.93 | Down |
| 97 | ZNF641 | 7962794 | 0.00046 | 1.12 | Up |
| 98 | ZNF763 | 8025978 | 0.04287 | 1.06 | Up |
