# Supplementary material for: Early-pregnancy transcriptome signatures of preeclampsia: from peripheral blood to placenta
Source: Sci Rep. 2020 Oct 12;10:17029. doi: 10.1038/s41598-020-74100-1 (PMC7550614; doi:10.1038/s41598-020-74100-1)
Supplement: Supplementary file 3 — Supplementary information 3. [file 41598_2020_74100_MOESM3_ESM.docx]

**Early-Pregnancy Transcriptome Signatures of Preeclampsia:**

**From Peripheral Blood to Placenta**

Aishwarya P. Yadama, BS^1^; Enrico Maiorino, PhD^1^; Vincent J. Carey, PhD^1^; Thomas F. McElrath, MD, PhD^2^; Augusto A. Litonjua, MD, MPH^3^; Joseph Loscalzo, MD, PhD^4^; Scott T. Weiss, MD, MS^1*^; Hooman Mirzakhani, MD, PhD, MMSc^1*^

**Supplementary File 3 (S3):**

**Additional Methods:**

- Study Cohorts
- Assessment of Preeclampsia and Risk Factors
- Whole Blood Gene Expression Profiling
- Closeness of risk factor modules to preeclampsia module in the interactome
- Closeness of risk factor modules to preeclampsia module in comparison to unrelated conditions

**Supplemental Figure S3A:** Degree distribution of risk factor modules and negative control conditions

**Supplemental Figure S3B:** Z-scores of the average distance between the risk factor modules and preeclampsia in comparison to z-scores of the average distance between the negative controls selected from DisGeNet and preeclampsia.

*Study Cohorts*

*I. Discovery cohort*

A subset of the participants from the VDAART was used for the discovery of peripheral blood transcriptomic variations related to preeclampsia (PE), maternal asthma, excess BMI and vitamin D insufficiency at early pregnancy. Details of the VDAART design and protocol as well as primary and secondary outcomes are published^1, 2, 3^. In brief, eligible participants in the VDAART were women between the ages of 18 and 39 years with the estimated gestational ages of 10 and 18 wga, who were non-smokers, had a history of physician-diagnosed asthma or atopy, or whose partner had a history of asthma or atopy. Pregnant women with known chronic hypertension were excluded from entry into the study population. **Supplemental File S1** provides additional information on the eligibility and exclusion criteria in the VDAART.

Participants of the discovery gene expression study comprised 157 subjects, chosen among VDAART participants, 47 of whom experienced PE. Overall, 67 pregnant women among participants in the VDAART included in the intent to treat analysis (N=816) developed PE^3^, of whom, 47 (70.14%) had a suitable RNA sample available at entry into the trial (10–18 wga) and were included in this study. Controls were chosen from the VDAART subjects with normal pregnancy courses matched for age (within 5 years), race, and study center, such that each subject had 2 matched controls. Once the initial set of matched study subjects and controls was formed, additional controls were incorporated to enhance the range of 25OHD concentrations present among the control subjects (total *N*_controls_= 110). Of these subjects, 63 (63 vs 94) had a history of diagnosed asthma, 119 (119 vs 38) with vitamin D insufficiency (≤30 ng/mL), and 104 (104 vs 53) with excess BMI (≥25 kg/m^2^) at enrollment.

*II. Replication cohorts*

A population selected to replicate the differentially expressed genes in peripheral blood associated with PE in the VDAART discovery cohort was obtained from a nested case-control study of PE (N cases= 16 and N controls= 16) within the OMEGA study, a prospective cohort study designed to assess dietary and other lifestyle risk factors of PE and other medical complications of pregnancy^4^. The participants of this study were adult nulliparous women, who initiated prenatal care prior to 20 wga at the Swedish Medical Center in Seattle, Washington, USA.^4^ The range of gestational weeks of participants’ peripheral blood samples from this study included in the replication analysis was 10-16.

A second group of the VDAART participants with maternal asthma but without a diagnosis of PE or gestational hypertension during their pregnancy and with suitable RNA samples from peripheral blood available at entry into the trial (10–18 wga) was used for the replication of gene signatures related to maternal asthma, excess BMI and vitamin D insufficiency. This group included peripheral blood samples from 10-18 wga of 24 pregnant women, of whom, 10 (10 vs 14) had a history of physician-diagnosed asthma, 17 (17 vs 7) with vitamin D insufficiency (≤30 ng/mL) and 14 (14 vs 10) with excess BMI (≥25 kg/m^2^).

*Preeclampsia Diagnosis*

*I. Discovery cohort: VDAART*

The occurrence of PE was a secondary outcome of the VDAART as well as an adverse event. After delivery, medical records were abstracted, and a committee of 4 board-certified obstetricians conducted a blinded review of 276 abstracted charts of subjects with a noted diagnosis of hypertension, proteinuria, or PE to determine PE status. The diagnosis of PE at the time of the record reviews was based on the definition of PE by the 2013 Task Force on Hypertension in Pregnancy^5^, which included the identification of high blood pressure (BP) and either proteinuria (≥300 mg per 24-hour collection or ≥1+ on a urine dipstick) or the presence of elevated liver enzymes, high platelet count, headache, or visual disturbances after 20 wga. High BP was diagnosed for participants who had a systolic BP at or above 140 mmHg, a diastolic BP at or above 90 mmHg or both, with a second elevated measurement, noted in the medical record at least 4 hours after the first measurement was taken.

*II. Replication cohort: OMEGA*

In the OMEGA replication cohort, PE cases were diagnosed according to the ACOG guidelines in the presence of both hypertension and proteinuria^4^. Women with a history of chronic hypertension and/or pre-gestational diabetes, as well as women with non-singleton pregnancies, had been excluded^4^. Controls had been selected among those women who had normotensive pregnancies uncomplicated by proteinuria or gestational diabetes.

*Assessment of Maternal Asthma, Vitamin D and BMI Status in VDAART*

The main risk variables of interest included the presence or absence of 1) maternal diagnosis of asthma, 2) BMI representing overweight to an obese range (≥25 kg/m^2^), and 3) vitamin D insufficiency (≤30 ng/mL).

1. *Assessment of maternal asthma*

Completed questionnaires at enrollment and monthly maternal health follow-up surveys thereafter provided information on the absence or presence of maternal asthma from enrollment. A subject was considered to have asthma if she reported physician-diagnosed asthma at any time in her life.

1. *Assessment of maternal vitamin D and BMI status*

Serum assays of 25OHD were performed at the Channing Division of Network Medicine, the data coordination center of the VDAART^2^. 25OHD levels were quantitatively determined using the FDA approved, direct, competitive chemiluminescence immunoassay (CLIA) on a DiaSorin LIAISON®25-OH Vitamin D Total machine. This assay is co-specific for 25-hydroxyvitamin D_3_ and 25-hydroxyvitamin D_2_; a total of 25OHD values including 25OHD_2_ and 25OHD_3_ measured by the above method were used for determining the variation of transcriptomes related to vitamin D status. For quality control, the laboratory used the US National Institute of Standards and Technology (NIST) level 1 protocol. Inter-and intra-assay CVs for this assay were 11.2% and 8.1%, respectively. Vitamin D insufficiency status was defined at a 25OHD threshold of <30 ng/mL based on the Endocrine Society’s recommendations ^6^ and prior observation in the relationship of pregnancy vitamin D status and adverse pregnancy outcomes including PE^3, 7^. Height and weight were obtained from the measurements in the VDAART participants’ first appointment (10-18 wga) and BMI was calculated using weight in kilograms divided by the square of height in meters.

*Whole Blood Gene Expression Profiling*

*RNA isolation*

*I. VDAART*

Total RNA was isolated from whole blood using the QIAGEN PAXgene Blood RNA Kit according to the manufacturer’s protocol. The GLOBINclear Kit (Ambion) was used to remove α and β globin mRNA from the samples to increase the sensitivity of the gene expression assays by improving the detection rate of expressed genes. The RNA was quantified using the Nanodrop 8000 and checked for high integrity before the preparation of cDNA. The integrity of RNA samples was assessed using the Agilent 2100 Bioanalyzer, and the purity of the samples was confirmed using the NanoDrop spectrophotometer and RNA integrity number (RIN ≥8). Gene expression was assessed using the Affymetrix Human Gene 1.0 ST Array. Biotinylated cRNA was prepared according to the manufacturer’s protocol, and hybridization was processed according to the protocol for the GeneChip Hybridization Control Kit.

*II. OMEGA*

In the OMEGA study, PAXgene Blood RNA Kit was used for extraction and purification of total RNA. The RNA concentration was measured by UV absorbance at 260nm using the NanDrop ND1000 spectrophotometer RNA samples with absorbance >2.0 at 260 nm and 280 nm readings indicating a high level of purity. The resultant cDNA from the prepared RNA was hybridized onto Affymetrix Human Genome U133 Plus 2.0 GeneChip Arrays. Data from each array was subsequently quantified using the GeneChip Operating software ^4^.

*Microarray Processing and Quality Control of Gene Expression Data*

1. *Discovery cohort in VDAART*

Gene expression of the discovery cohort in the VDAART subjects was profiled using the Affymetrix GeneChip. After the production and processing of images, an expression set with 33,297 probes and 157 samples, collected at 10 to 18 wga, was generated, representing the 47 subjects with PE and the 110 controls. We checked the quality of the arrays by examining quantiles before and after normalization. Background adjustment, log_2_ transformation, and quantile normalization were performed using the robust multiarray analysis (“rma”) function in R BioConductor’s “affy” library to minimize non-biological variability among arrays. Principal component analysis (PCA) of samples was done to identify any extreme outliers. Thereafter, we merged phenotypes of interest with the expression set. Probes were then annotated using the annotation package for Bioconductor pd.hugene.1.0.st.v1 and subsequently extracted probes of autosomal chromosomes (19,137 probes) which we used in our analysis. The interquartile range (IQR) filter (values of IQR including 80% of the probe expressions) was implemented in R using the “genefilter” package from Bioconductor to remove expressions showing little changes within arrays. To remove all unwanted sources of variation while protecting the contrasts due to the primary variables of interests (PE, maternal asthma, vitamin D and BMI status), we applied Surrogate Variable Analysis (SVA) using the “sva package” from Bioconductor to account for confounders (i.e., treatment arm, batch effect) and additional sources of expression heterogeneity. This process resulted in an expression set of 15,309 probes belonging to 157 samples collected at 10 to 18 weeks of pregnancy, representing 47 PE cases and their 110 matched controls.

*II. Replication Cohorts*

*IIA: OMEGA cohort*

Gene expression of OMEGA subjects was profiled using Affymetrix GeneChip Arrays. Quantiles of raw expression and principal components (PCs) across arrays were examined before and after background adjustment normalization and log2 using the “rma” function in the R “affy” library. The quality of arrays in AffyBatch object was assessed by running the “QCReport” function in the R library’s “affyQCReport.” PCA was additionally conducted to identify any extreme outliers among samples. Expression data for 2 of the samples were identified as outliers and removed, and quantile-normalized and background-adjusted expression data were generated and reexamined using PCA. The results were also reexamined by running the “QCReport” function. The microarray expression data were annotated using “hgu133plus2,” available on Bioconductor. The annotated expression from autosomal chromosomes was included in the analysis. To capture the expression heterogeneity due to unknown and unmeasured factors and retain the signal due to the primary variables of interest, surrogate variable analysis (SVA) was conducted by applying the SVA function to the expression data. The final expression data set contained 40,003 rows of background-adjusted, log2-transformed, rma-summarized, and quantile-normalized expression data for 14 PE cases and 16 controls.

*IIB: Replication cohort in the VDAART*

The second group in the VDAART that was used for replication of gene signatures associated with maternal asthma, vitamin D and BMI status included 24 pregnant women with available RNA samples and without PE diagnosis from peripheral blood at 10-18 wga. Gene expression of the replication cohort in the VDAART subjects was profiled using the Affymetrix GeneChip platform. The qualified RNAs from these subjects were processed to create an expression set of 33,297 gene probes. Next, we background adjusted, log_2_ transformed, and quantile normalized the arrays by applying the robust multiarray analysis (“rma”) function in R BioConductor’s “affy” library and conducted PCA on the samples to check for extreme outliers. Next, we created a subset of the expression set to include only annotated probes of autosomal chromosomes and applied the interquartile range (IQR) filter from R Bioconductor “genefilter” package to remove expressions with variance less than 20% within arrays (values of IQR including 80% of the probe expressions). To remove all unwanted sources of variation while protecting the contrasts due to the variables of interest (maternal asthma, vitamin D and BMI status), we applied SVA to account for confounders (i.e., batch effect) and additional sources of expression heterogeneity. The final expression data set contained 15,222 probes of background-adjusted, log_2_-transformed, rma-summarized, and quantile-normalized expression data for 24 subjects, of whom 10 had a history of maternal asthma, 17 had vitamin D insufficiency (≤30 ng/mL), and 14 had excess BMI (≥25 kg/m^2^).

**Closeness of risk factor modules to preeclampsia module in the interactome**

To test whether the PE module was close and connected to gene signatures related to its risk factors, i.e., maternal asthma, excess BMI and vitamin D insufficiency, we measured the closeness between the four sets of gene signatures (modules) and compared the obtained parameters to the one from a random expectation of closeness in the interactome. The assumption behind this exercise was that even if the genes in the modules of the risk factors do not overlap with the PE module, they should be localized in the same neighborhood as the PE module in the interactome. The closeness was computed as the average of all possible shortest paths between two sets of genes ^8^:

In this formula, d(s, t) is the shortest path from a gene “s” in the signature module (Pm) to a gene “t” of the Pathway (Pw), and and denote the number of genes in each set. To determine the significance of this quantity, we randomly generated 1000 degree-preserving samples of the PE module as well as those of maternal asthma, excess BMI and vitamin D insufficiency and all the Pathways of interest in the modules. For each node set of size *n*, the degree-preserving randomization extracted *n* nodes with the same degree distribution as the original set. To avoid genes with unique degree values being infrequently selected, degrees were binned in intervals of increasing sizes.

For each pair of gene sets, we calculated all of the average distances between their corresponding random samples, obtaining 1000 random values of average distance ^8^. The significance was evaluated by computing the z-score:

In this formula and denote the mean value and standard deviation of the random average distance*.* Any distance with a z-score less than -1.65 (i.e., modules closer than expected) was deemed significant; as such a cutoff corresponds to a *P-*value≈0.05 in the case of a normally distributed variable. To test the specificity of our observations in the PPI, we also compared the closeness of risk factor modules (asthma, BMI and vitamin D) to the preeclampsia module in comparison to unrelated conditions.

**Closeness of risk factor modules to preeclampsia module in comparison to unrelated conditions**

To test the specificity of our observations (i.e., the closeness of the risk factor modules to preeclampsia modules in the PPI), we selected several gene sets associated with phenotypes that are unrelated to PE. These negative controls are useful to account for potential biases in the network topology caused by technical and biological factors, such as literature bias towards most commonly studied disease genes and non-specificity of the biological processes shared among most diseases (e.g. inflammation). We used the curated gene-disease associations (GDAs) on the DisGeNET platform (http://www.disgenet.org/)^9^ as the main source for the analysis and selected the candidate phenotypes as described below. We first filtered all the GDAs that were not classified as “Disease or Syndrome” or involving genes not mapped to the PPI network, obtaining 5756 unique genes and 7357 unique phenotypes. Since the shortest path distance in a network is influenced by node degree, we restricted the pool of candidate phenotypes to only those with a similar average degree of the associated genes to the average degree in the risk factor gene sets (i.e., asthma, BMI and vitamin D gene signatures). Specifically, since all the three risk factor gene sets approximately had a similar average degree in the modules (113, 106 and 111, respectively), we considered the average of the average degrees in risk factor modules as a reference point (~110). We ranked all the phenotypes by the absolute deviation of their average degree from the reference degree, that is

$$\text{Degree of deviation}=|\bar{d}_{i}-\bar{d}_{REF}|$$

Where $\bar{d}_{i}$ is the average degree of the genes associated with candidate phenotype *i* and $\bar{d}_{REF}$ is the average of the average degrees of the risk factor modules. We then selected the 30 phenotypes with the lowest degree deviation and at least 30 annotated genes (**Figure S3.1**). Almost all but 3 of the considered phenotypes in the list were annotated to genes with a higher average degree than the risk factor modules, probably owing to the origin of both STRINGdb protein-protein interactions and DisGeNet gene-disease associations from literature sources (**Figure S3.1**). However, the largest positive degree deviation in the top 30 phenotypes was below 60, allowing us to perform a strict but still fairly balanced worst-case comparison between the modules. To further avoid the test to be affected by the degree differences between individual genes in the modules, we considered the z-scores of the average distances as the unit of comparison, using the same procedure described in the method section^8^. As shown in **Figure S3.2**, the risk factor modules (Asthma, BMI and vitamin D status) are considerably closer to PE module than any of the random phenotypes, suggesting that the pathobiological responses related to the risk factors share specific molecular processes that cannot be attributed to general connectivity patterns between disease genes in the interactome.

**Figure S3A**. Degree distribution of risk factor modules (blue) and negative controls (orange). The bars on top indicate the average degree (light red) and median degree (dark red) of each gene set.

**Figure S3B**. Negative values of the z-scores are shown for clarity. (blue bars) negative z-scores of the average distance between the risk factor modules and PE; (orange bars) negative z-scores of the average distance between the negative controls selected from DisGeNet and PE.

**References**

1. Litonjua AA*, et al.* Effect of Prenatal Supplementation With Vitamin D on Asthma or Recurrent Wheezing in Offspring by Age 3 Years: The VDAART Randomized Clinical Trial. *JAMA* **315**, 362-370 (2016).

2. Litonjua AA*, et al.* The Vitamin D Antenatal Asthma Reduction Trial (VDAART): rationale, design, and methods of a randomized, controlled trial of vitamin D supplementation in pregnancy for the primary prevention of asthma and allergies in children. *Contemp Clin Trials* **38**, 37-50 (2014).

3. Mirzakhani H*, et al.* Early pregnancy vitamin D status and risk of preeclampsia. *J Clin Invest* **126**, 4702-4715 (2016).

4. Enquobahrie DA, Qiu C, Muhie SY, Williams MA. Maternal peripheral blood gene expression in early pregnancy and preeclampsia. *Int J Mol Epidemiol Genet* **2**, 78-94 (2011).

5. American College of O, Gynecologists, Task Force on Hypertension in P. Hypertension in pregnancy. Report of the American College of Obstetricians and Gynecologists' Task Force on Hypertension in Pregnancy. *Obstet Gynecol* **122**, 1122-1131 (2013).

6. Holick MF*, et al.* Evaluation, treatment, and prevention of vitamin D deficiency: an Endocrine Society clinical practice guideline. *J Clin Endocrinol Metab* **96**, 1911-1930 (2011).

7. Wei SQ. Vitamin D and pregnancy outcomes. *Curr Opin Obstet Gynecol* **26**, 438-447 (2014).

8. Sharma A*, et al.* Integration of Molecular Interactome and Targeted Interaction Analysis to Identify a COPD Disease Network Module. *Sci Rep* **8**, 14439 (2018).

9. Pinero J*, et al.* The DisGeNET knowledge platform for disease genomics: 2019 update. *Nucleic Acids Res* **48**, D845-D855 (2020).
